# The T300A Crohn's disease risk polymorphism impairs function of the WD40 domain of ATG16L1

**DOI:** 10.1038/ncomms11821

**Published:** 2016-06-08

**Authors:** Emilio Boada-Romero, Inmaculada Serramito-Gómez, María P. Sacristán, David L. Boone, Ramnik J. Xavier, Felipe X. Pimentel-Muiños

**Affiliations:** 1 Instituto de Biología Molecular y Celular del Cáncer (CSIC-Universidad de Salamanca), Centro de Investigación del Cáncer, Campus Miguel de Unamuno, Universidad de Salamanca, Salamanca 37007, Spain; 2 Departments of Microbiology and Immunology, Indiana University School of Medicine-South Bend, South Bend, Indiana 46617, USA; 3 Center for Computational and Integrative Biology, Massachusetts General Hospital, Harvard Medical School, Boston, Massachusetts 02114, USA; 4 Broad Institute of MIT and Harvard, Cambridge, Massachusetts 02142, USA

## Abstract

A coding polymorphism of human *ATG16L1* (rs2241880; T300A) increases the risk of Crohn's disease and it has been shown to enhance susceptibility of ATG16L1 to caspase cleavage. Here we show that T300A also alters the ability of the C-terminal WD40-repeat domain of ATG16L1 to interact with an amino acid motif that recognizes this region. Such alteration impairs the unconventional autophagic activity of TMEM59, a transmembrane protein that contains the WD40 domain-binding motif, and disrupts its normal intracellular trafficking and its ability to engage ATG16L1 in response to bacterial infection. TMEM59-induced autophagy is blunted in cells expressing the fragments generated by caspase processing of the ATG16L1-T300A risk allele, whereas canonical autophagy remains unaffected. These results suggest that the T300A polymorphism alters the function of motif-containing molecules that engage ATG16L1 through the WD40 domain, either by influencing this interaction under non-stressful conditions or by inhibiting their downstream autophagic signalling after caspase-mediated cleavage.

## Abstract (web summary)

The T300A substitution in ATG16L is associated with Crohn's disease risk and disrupts clearance of intracellular pathogens by autophagy. Here the authors show that the mutation impairs interaction of ATG16L with TMEM59 and disrupts unconventional TMEM-induced autophagy, an aspect of innate immunity.

## Graphical abstract

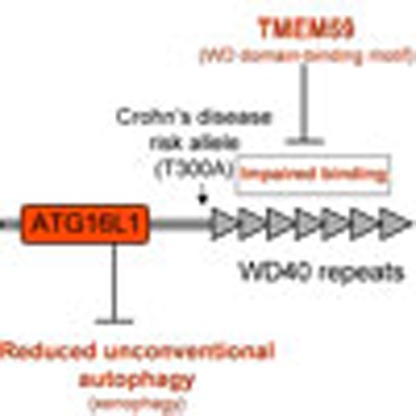

Crohn's disease (CD) is a complex genetic and multifactorial condition that involves inflammation of discontinuous areas of the small intestine and colon^1^,^2^,^3^. The pathology is initially episodic, but usually evolves to chronic and refractory states, causing severe complications that may require resective surgery^4^,^5^. The underlying molecular causes of CD have remained elusive.

A number of genetic association studies have contributed to the identification of molecules and signalling pathways involved in the onset of this condition^6^,^7^,^8^,^9^,^10^. Several coding risk polymorphisms identified in such studies correspond to molecules that mediate innate immunity and autophagy, suggesting a role for these processes in the disease^3^,^4^,^11^. Autophagy is an intracellular degradation pathway that helps maintain the metabolic homeostasis of the cell^12^,^13^,^14^. A selective form of autophagy, termed xenophagy, plays an important role in innate immunity by promoting the elimination of invading microorganisms^15^,^16^. Thus, different mechanisms mediate sequestration of infectious agents into autophagic vesicles that eventually fuse with lysosomes for degradation of their contents^17^,^18^,^19^,^20^. CD risk mutations involving the innate immune receptor NOD2 lead to an inability to recognize intracellular microorganisms, defective downstream xenophagy and poor defence against infection^21^. In addition, a risk polymorphic form of the critical autophagic effector ATG16L1 (T300A) impairs the autophagic clearance of intracellular pathogens^3^,^4^. The current paradigm is that dysfunctional xenophagy and altered innate immunity caused by the defective alleles of these two molecules prevents proper control of the commensal intestinal flora and favours the onset of inflammation and CD (refs 4, 22, 23).

ATG16L1 mediates autophagy by assembling the molecular complex that lipidates LC3 (ref. 24), the main marker of autophagic vesicles^25^. In this complex, the dimer ATG5-ATG12 holds the lipidating activity^26^, whereas ATG16L1 defines the site where LC3 will be lipidated^24^. ATG16L1 includes a C-terminal domain formed by seven WD40-type repetitions (the WD domain, WDD; residues 320–607)^27^, the function of which has remained unclear. This region is absent in yeast Atg16, indicating that it is not necessary for the basic functions of autophagy in unicellular organisms^27^. In fact, it appears to be irrelevant for basal or starvation-induced autophagy in mammalian cells, as ATG16L1-deficient cells fully recover these canonical autophagic activities when restored with a version of ATG16L1 that lacks the WD40 repeats^28^,^29^,^30^,^31^. Such observation is in line with the fact that most of the critical autophagic effectors that bind ATG16L1 recognize regions other than the WDD, like, for example, ATG5 (which recognizes residues 1–79 of ATG16L1)^27^, FIP200 (residues 230–250 and 288–300 (ref. 28) or 229–242 (ref. 29), WIPI2 (residues 207–230)^32^ and Rab33 (residues 80–265)^33^. Therefore, the function of the WD40 repeats is likely unrelated to canonical autophagy. Instead, it might be related to the various alternative activities recently linked to ATG16L1 through the study of ATG16L1-deficient experimental systems, like inflammatory control^34^,^35^, trafficking of secretory vesicles in Paneth cells^36^,^37^,^38^ or some forms of xenophagy^36^,^39^,^40^.

Recent work from our laboratory showed that the WDD is specifically recognized by a novel 19-amino acid element found in the intracellular domain of the transmembrane protein TMEM59 (residues 263–281) as well as other molecules^41^. TMEM59 is a glycosylated, widely expressed, type-I transmembrane protein that has been involved in regulating the complex glycosylation of the amyloid precursor protein^42^. We previously reported that TMEM59 is able to engage ATG16L1 to induce an unconventional autophagic process that causes LC3 labelling of the same single-membrane endosomes where TMEM59 is located, thus promoting a more efficient lysosomal targeting of these vesicles^41^. This activity requires four critical positions within the active element (Y268, E272, Y277 and L280) that define a novel motif able to bind the WDD (ref. 41). Such unconventional autophagic event participates in the LC3 labelling of single-membrane phagosomes containing *Staphylococcus aureus*, suggesting a role in the innate cellular response against invading microorganisms^41^. The WDD of ATG16L1 has also been shown to recognize ubiquitin^43^, an ability that might be common to functionally diverse WD40 domain folds^44^.

How the T300A mutation alters the biology of ATG16L1 has remained unclear. Existing evidence argues that it does not impair, or has relatively minor effects, on basal and starvation-induced autophagy^30^,^39^,^45^. Instead, it appears to derail some of the alternative functions carried out by ATG16L1, such as maintenance of the secretory compartment in intestinal Paneth and goblet cells^38^,^46^, inflammation control^34^ and xenophagy^39^,^45^,^46^,^47^,^48^,^49^. Recently, it has been shown that T300A increases susceptibility of ATG16L1 to caspase-3 cleavage in a neighbouring consensus site, leading to decreased levels of full-length ATG16L1 in the context of a stressful situation and, consequently, dysfunctional autophagy and xenophagy^46^,^47^.

Here we show that, under non-stressful conditions, unprocessed ATG16L1-T300A (hereafter ATG16L1-A300) has an intrinsic reduced ability to bind certain versions of the ATG16L1-binding motif that recognizes the WDD, an alteration that results in defective downstream autophagy, slow intracellular trafficking of motif-containing transmembrane molecules and reduced engagement of ATG16L1 in response to *S. aureus* infection.

## Results

### ATG16L1 WDD is required for TMEM59-induced autophagy

**Figure 1:**
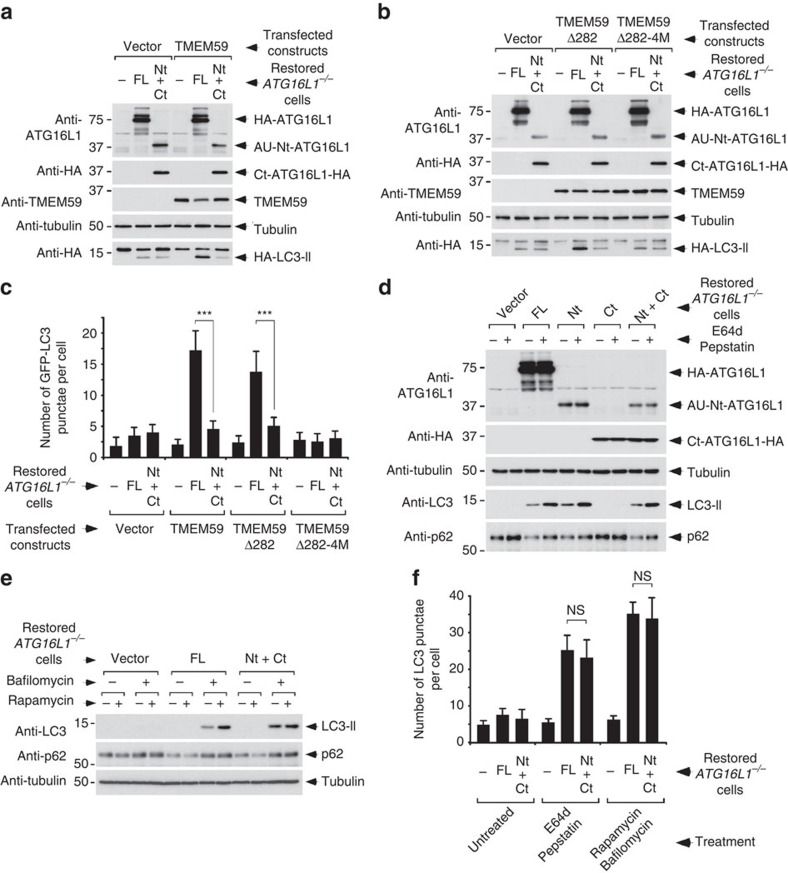
Impaired TMEM59-induced autophagy in the presence of ATG16L1 fragments resulting from caspase-3-mediated cleavage. (**a**,**b**) Immunoblot analysis of HA-LC3 lipidation induced by TMEM59 overexpression for 36 h in *ATG16L1*^−/−^ HCT116 cells restored with full-length HA-ATG16L1 (FL), both ATG16L1 fragments (Nt: 1–299; Ct: 300–607) or irrelevant vector (−). TMEM59-Δ282 is the largest C-terminal deletion that retains the autophagic potential of the molecule. 4M designates a mutated form of TMEM59 where the four residues that are essential for its autophagic activity are mutated to alanine. (**c**) Quantification of GFP-LC3 punctae per transfected cell induced by TMEM59 overexpression for 36 h in the same cell lines as in **a** and **b**. Shown are mean values±s.d. (*n*=50 cells, ***P<0.001 Student's *t*-test). (**d**,**e**) Immunoblot analysis of endogenous LC3 lipidation and p62 expression levels induced in the indicated restored *ATG16L1*^*−/−*^ HCT116 cells by treatment with E64d/Pepstatin (10 μg ml^−1^ each, 8 h) or bafilomycin (50 nM, 8 h)±rapamycin (2 μg ml^−1^, 8 h). (**f**) Quantification of endogenous LC3 punctae per cell induced by the indicated treatments (treatment conditions were as in **d** and **e**; NS, not significant, *P*>0.05 Student's *t*-test). All results shown in this figure are representative of at least two repetitions.

To evaluate the functional consequences of caspase-3-mediated processing of ATG16L1 on the activity of molecules that include a WDD-binding motif (for example, TMEM59), we reconstituted *ATG16L1*^−/−^ HCT116 cells^45^ with either intact ATG16L1, the N-terminal or C-terminal portions that result from caspase-3 processing, or both portions simultaneously. We then subjected the resulting cellular strains to TMEM59-induced autophagy or autophagy promoted by conventional stimuli. Interestingly, the ability of TMEM59 to induce lipidation of co-transfected HA-LC3 was blocked by uncoupling of the N- and C-terminal domains of ATG16L1 (Fig. 1a). However, clearance of p62-HA remained unaltered in the same conditions (Supplementary Fig. 1a), suggesting that TMEM59-induced autophagy and the basal autophagic flux have different ATG16L1 domain requirements. The autophagic activity of TMEM59 in this setting was restricted to the functional motif that we previously identified in its intracellular domain, since it was retained by a deleted version of the protein exclusively harbouring the 19-amino acid minimal active peptide (TMEM59-Δ282)^41^ and blocked by mutation of the four critical residues to alanine (4M version^41^; Fig. 1b). Inhibition of TMEM59-induced autophagy by split ATG16L1 was also detected by measuring the translocation of co-transfected green fluorescent protein (GFP)-LC3 (Fig. 1c) or endogenous LC3 (Supplementary Fig. 1b) to a punctated pattern. Notably, basal and rapamycin-induced autophagy proceeded normally provided that the N-terminal portion of ATG16L1 was present, regardless of whether or not it was physically connected to the C-terminal region (Fig. 1d–f). This is consistent with previous reports showing that the N-terminal domain of ATG16L1 suffices to sustain basal autophagy in mammalian cells^28^,^29^,^30^,^31^. Taken together, these results argue that ATG16L1 includes two functional modules: an N-terminal effector region that mediates all autophagic activities of the protein, and a C-terminal WD40 domain that acts as a docking site for upstream inducers of unconventional autophagy, such as TMEM59. In addition, these data suggest that caspase-cleaved ATG16L1 may preferentially impair the autophagic activity of molecules that engage ATG16L1 through the WDD.

### ATG16L1-A300 impairs TMEM59-induced autophagy

**Figure 2:**
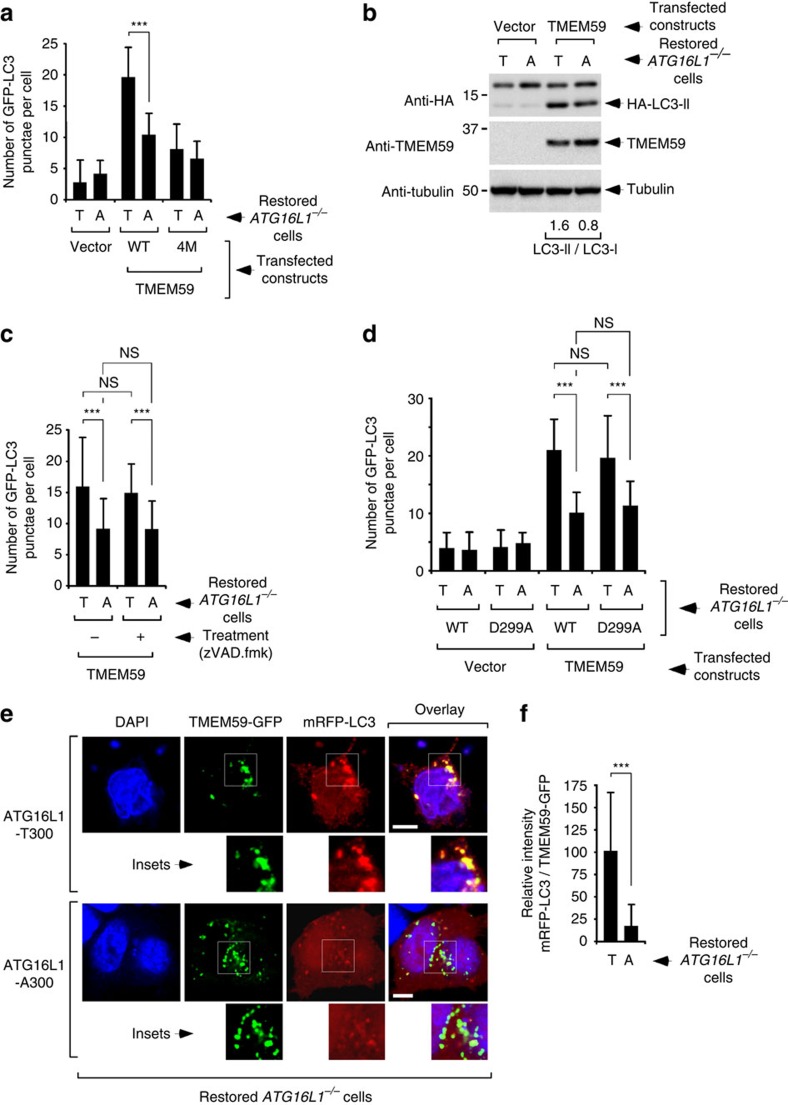
Defective autophagic function of TMEM59 in the presence of the ATG16L1-A300 Crohn's disease risk allele. (**a**) Quantification of GFP-LC3 punctae per transfected cell induced by TMEM59 overexpression for 24 h in *ATG16L1*^−/−^ HCT116 cells restored with HA-ATG16L1-T300 (T) or HA-ATG16L1-A300 (A). Shown are mean values±s.d. (*n*=50 cells, ***P<0.001 Student's *t*-test). (**b**) Immunoblot analysis of HA-LC3 lipidation induced by TMEM59 overexpression in the same conditions and cell lines as in **a**. The LC3-II/LC3-I signal intensity ratio is shown at the bottom of the relevant lanes. (**c**,**d**) Quantification of GFP-LC3 punctae per transfected cell induced by TMEM59 overexpression for 36 h in the same cell lines as in **a**, and in the absence or presence of zVAD.fmk (**c**, 50 μM for the last 8 h of culture), or in *ATG16L1*^−/−^ HCT116 cells restored with the indicated ATG16L1 mutants (**d**). Data are displayed as in **a** (NS, not significant, *P*>0.05 Student's *t*-test; ***P<0.001 Student's *t*-test). (**e**) Representative confocal pictures showing colocalization between TMEM59-GFP and mRFP-LC3 co-expressed for 36 h in the same cell lines as in **a**. Scale bars represent 5 μm. (**f**) Quantification of the intensity of mRFP-LC3 present in TMEM59-GFP-positive vesicles (mean±s.d., *n*=100 vesicles present in 8 cells, ***P<0.001 Student's *t*-test; right panel). Data are presented as a fraction of the mean value obtained in cells expressing ATG16L1-T300. All displayed results are representative of at least two independent repetitions.

Recent reports have shown that enhanced processing of ATG16L1-A300 by caspase 3 occurs in response to stressful inducers of autophagy, such as bacterial infection or metabolic stress^46^,^47^. However, given that position 300 localizes in the vicinity of the WD40 repeats (residues 320–607), we wondered whether the A300 risk polymorphism might influence the unconventional autophagic activities mediated by the WDD under homeostatic conditions with minimal caspase 3 activity. Using *ATG16L1*^−/−^ HCT116 cells restored with T300 or A300 forms of ATG16L1 (Supplementary Fig. 2a), we found that TMEM59-induced autophagy was impaired in cells expressing the A300 allele, both at the level of LC3 translocation to a vesiculated pattern (Fig. 2a; Supplementary Fig. 2b) and, less prominently, by measuring LC3 lipidation (Fig. 2b). Again, the levels of co-transfected p62-HA remained unchanged (Supplementary Fig. 2c), suggesting that the autophagic activity of TMEM59 that becomes derailed by ATG16L1-A300 is different from the basal autophagic flux. Consistently, no alterations in basal or rapamycin-induced autophagy were detected in cells harbouring the risk allele (Supplementary Fig. 2d–f). Importantly, the decreased autophagic activity of TMEM59 caused by ATG16L1-A300 occurred even in the presence of a pan-caspase inhibitor (Fig. 2c) that blocks caspase-3 activation and ATG16L1 cleavage induced by tumour necrosis factor (TNF; Supplementary Fig. 3a). In addition, cells expressing a mutated version of ATG16L1-A300 (Supplementary Fig. 2a) that is insensitive to caspase cleavage (ATG16L1-A300-D299A; Supplementary Fig. 3b) displayed similar autophagic defects compared with caspase-sensitive ATG16L1-A300 (Fig. 2d). Control experiments showed no overt influence of the double mutant on basal or nutritional autophagy (Supplementary Fig. 2d–f). These results indicate that the autophagic defects displayed by TMEM59 in the presence of the risk allele do not require caspase processing of ATG16L1-A300.

Our previous studies in a variety of cell lines indicated that the autophagic activity of TMEM59 is directed against the same intracellular vesicles where this protein is located^41^, and this was also the case in HCT116 cells (Fig. 2e). In a series of confocal quantification experiments, we found that the vesicles harbouring TMEM59-GFP were decorated on average with less monomeric RFP (mRFP)-LC3 in HCT116 cells expressing the risk allele compared with those restored with the T300 form (Fig. 2e,f). These results indicate that ATG16L1-A300 impairs the ability of TMEM59 to induce the autophagic labelling of its own vesicular compartment.

### The A300 allele alters TMEM59 intracellular trafficking

**Figure 3:**
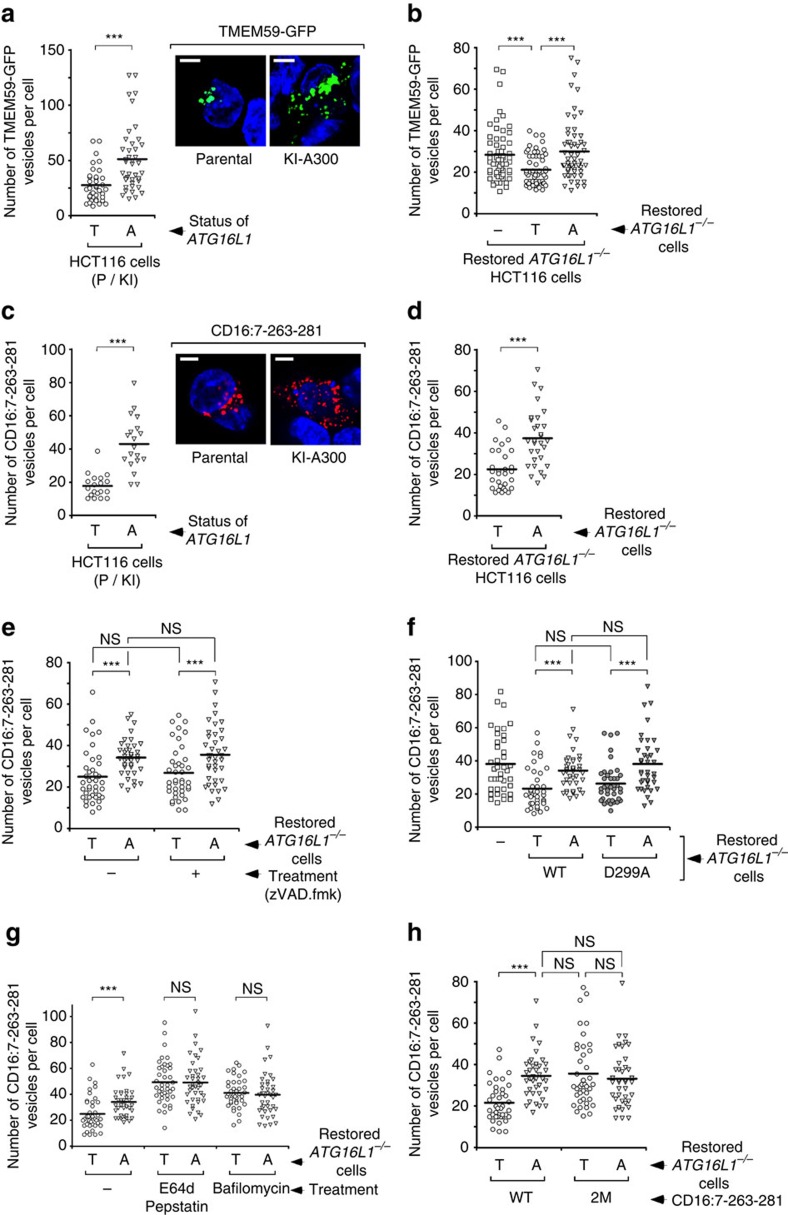
Altered intracellular trafficking of TMEM59 in cells expressing ATG16L1-A300. (**a**,**b**) Quantification of the number of TMEM59-GFP-positive vesicles per cell 24 h after transfection in parental (T) and ATG16L1-A300 knock-in (KI; A) HCT116 cell lines (**a**, *n*=40 cells), or in *ATG16L1*^−/−^ HCT116 cells restored with T300 (T) or A300 (A) forms of ATG16L1, or irrelevant vector (−), (**b**, *n*=60 cells). Shown are scatter plots where each event represents the score obtained for one cell and the thick horizontal line indicates the mean value (***P<0.001 Student's *t*-test). The inset shows representative confocal pictures; scale bars represent 5 μm. (**c**,**d**) Quantification of the number of intracellular vesicles containing aggregated, endocytosed CD16:7–263–281 chimera per cell after aggregation with anti-CD16 antibodies in the cellular strains shown in **a** and **b**, respectively (**c**, *n*=20 cells; **d**, *n*=30 cells). ***P<0.001 Student's *t*-test. The inset shows representative confocal pictures; scale bars represent 5 μm. (**e**–**h**) Quantification of the number of intracellular vesicles containing aggregated, endocytosed CD16:7–263–281 chimera in *ATG16L1*^−/−^ HCT116 cells restored with the indicated ATG16L1 constructs, (**e**) in the absence or presence of zVAD.fmk (50 μM, last 8 h of culture; *n*=40 cells), (**f**) in cells restored with caspase-3-sensitive and -insensitive (D299A) ATG16L1 constructs (*n*=40 cells), (**g**) in the absence or presence of E64d/pepstatin (10 μg ml^−1^ each, 8 h) or bafilomycin (50 nM, 8 h; *n*=40 cells) or (**h**) after aggregation of wild type or Y268A,E272A (2M) versions of the CD16:7–263–281 chimera (*n*=40 cells). ***P<0.001 Student's *t*-test; NS, not significant, *P*>0.05 Student's *t*-test. All shown results are representative of at least two independent repetitions.

Unexpectedly, we observed that the intracellular trafficking of TMEM59 was altered in cells expressing the A300 allele. Thus, these cells exhibited an increased number of TMEM59-GFP-positive vacuoles compared with those expressing ATG16L1-T300, both in HCT116 cells engineered to harbour the T300A mutation (*knock-in* (KI)-HCT116-A300 (ref. 45); Fig. 3a) and in *ATG16L1*^*−/−*^ HCT116 cells restored with HA-ATG16L1-A300 (Fig. 3b). This is likely a loss-of-function phenotype, since *ATG16L1*^−/−^ cells displayed the same anomaly (Fig. 3b). A similar defect was observed on forced aggregation of a chimeric transmembrane molecule containing the minimal active element of TMEM59 (CD16:7-263–281)^41^ with anti-CD16 antibodies (Fig. 3c,d), indicating an involvement of the ATG16L1-binding peptide. This result also argues that the trafficking alteration primarily affects the endocytic route, not the maturation pathway that exports TMEM59 from the endoplasmic reticulum to the plasma membrane. Again, neither zVAD.fmk (Fig. 3e) nor ATG16L1-A300-D299A (Fig. 3f and Supplementary Fig. 4a) prevented this phenotype, indicating that it does not require caspase processing of the A300 allele.

Given our previous results showing that TMEM59-positive endosomes recruit ATG16L1 and become labelled with LC3 to be more efficiently targeted for lysosomal degradation^41^, we investigated whether the accumulation of vesicles in the presence of the risk allele was due to a slower transit through the endocytic route. Treatment with lysosomal inhibitors normalized the number of vacuoles carrying CD16:7-263–281 (Fig. 3g) or TMEM59-GFP (Supplementary Fig. 4b) in cells expressing ATG16L1-T300 compared with those harbouring the risk form, suggesting that the observed accumulation was not due to an increased supply of vesicles to the endocytic route (that is, enhanced endocytosis) promoted by the A300 allele. In addition, a mutated version of the motif that blocks the autophagic activity but leaves the embedded YXXL endocytosis motif intact (Y268A,E272A; 2M)^41^ recapitulated the defective trafficking phenotype of CD16:7-263–281 in the presence of the T300 non-risk form (Fig. 3h). Conversely, it had no effect in ATG16L1-A300-expressing cells (Fig. 3h), arguing that the double mutation (2M) and ATG16L1-A300 cause redundant alterations. Together, these results suggest that poor LC3 labelling of the endosomes where TMEM59 is located slows down their transit along the endocytic route in cells expressing ATG16L1-A300, thus causing the observed vesicle accumulation. In line with this idea, the expression levels of transfected TMEM59 were often higher in the presence of defective ATG16L1, whether this defect came from uncoupling the N- and C-terminal domains of the molecule (see TMEM59 immunoblot in Fig. 1a) or was caused by the T300A mutation (Fig. 2b).

### Defective binding of ATG16L1-A300 to TMEM59

**Figure 4:**
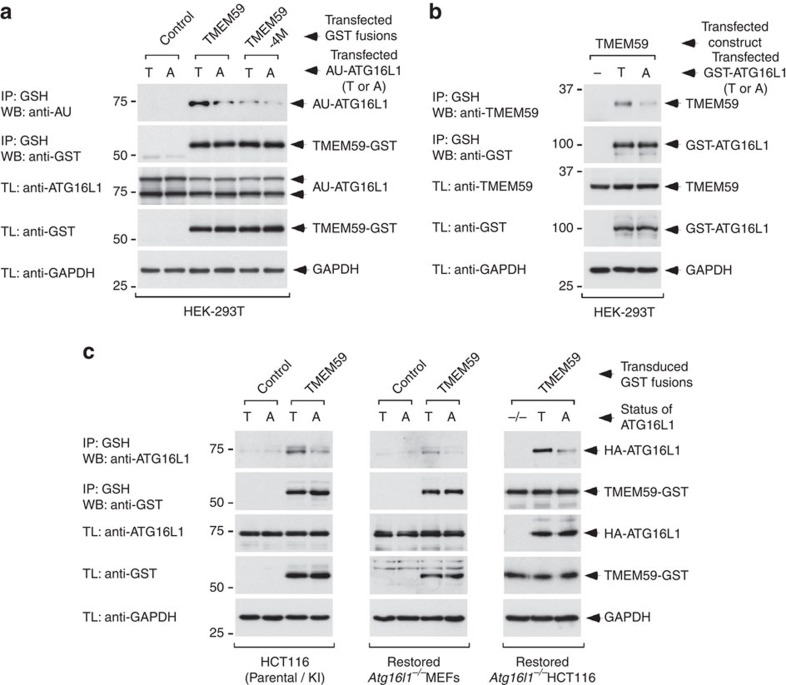
Impaired binding between TMEM59 and ATG16L1-A300 in co-precipitation assays. (**a**,**b**) Immunoblot analysis of agarose-glutathione (GSH) immunoprecipitates (IP) or total cell lysates (TL) from HEK-293T cells transfected with the indicated constructs and lysed 36 h after transfection. (**c**) Immunoblot analysis of GSH immunoprecipitates (IP) or whole-cell lysates (TL) of the indicated cell strains retrovirally transduced with TMEM59-GST for 36 h. All displayed results are representative of at least three repetitions.

Since the functional alterations introduced by ATG16L1-A300 in the autophagic activity and trafficking of TMEM59 are not prevented by caspase-3 inhibition (Figs 2c and 3e) or expression of a caspase-3-insensitive ATG16L1-A300 construct (ATG16L1-A300-D299A; Figs 2d and 3f and Supplementary Fig. 4a), they likely result from pathological mechanisms other than the reported increased susceptibility of the risk allele to caspase cleavage^46^,^47^. One possibility is that the A300 polymorphism alters the ability of the WDD to interact with its target motif. To test this idea we performed co-immunoprecipitation studies after expression of the relevant partners. We found that TMEM59 tagged with glutathione-S-transferase (GST; TMEM59-GST) co-precipitated less efficiently with the A300 variant of full-length ATG16L1 in transfected HEK-293T cells (Fig. 4a). The detected interaction was mediated by the WDD-binding motif, since it was inhibited by mutation of the four critical residues (4M version; Fig. 4a). In addition, less TMEM59 was found in GST-ATG16L1-A300 precipitates (Fig. 4b). Similar results were obtained in HCT116 cells engineered by knock-in to express ATG16L1-A300, and in *Atg16l1*^−/−^ mouse embryonic fibroblasts (MEFs)^36^ or *ATG16L1*^−/−^ HCT116 cells restored with T300 or A300 versions of ATG16L1 (Fig. 4c). Restored MEFs showed comparable levels of both ATG16L1 forms (Supplementary Fig. 5a), and unaltered basal autophagy in the presence of ATG16L1-A300 (Supplementary Fig. 5b). These data suggest that the T300A mutation impairs the ability of the WDD to interact with its natural ligands. However, given that the intracellular trafficking of TMEM59 is abnormal in ATG16L1-A300-expressing cells (Fig. 3), such impaired binding might be explained by an altered subcellular localization of any of the partners (TMEM59-GST or HA-ATG16L1 itself). To clarify this issue, we resorted to *in vitro* systems based on peptide arrays or pull-down co-precipitation assays involving recombinant proteins.

**Figure 5:**
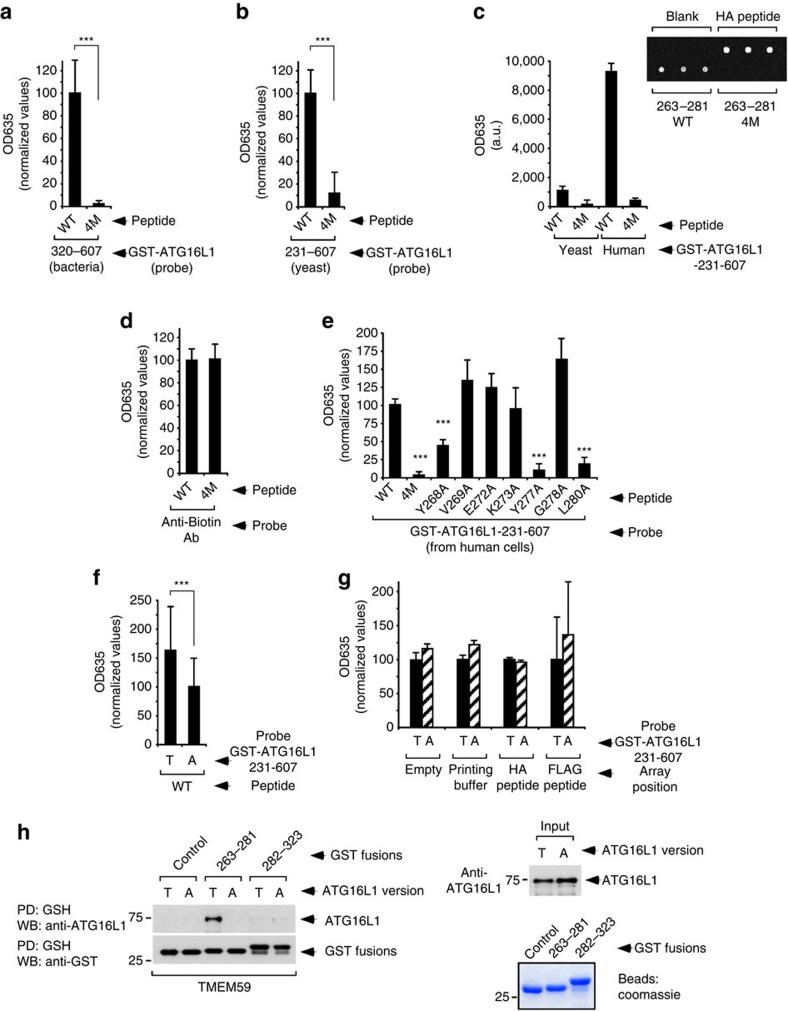
Defective *in vitro* binding between the active peptide 263–281 and ATG16L1-A300. (**a**–**g**) Quantification of binding signals provided by peptide microarrays developed with different ligands. Figures show mean values±s.d. (*n*=9 replicas, ***P<0.001 Student's *t*-test), unless otherwise indicated. (**a**,**b**,**d**,**e**) Values are expressed as a fraction of the figures provided by the wild type (WT) peptide. (**a**) Binding of GST-ATG16L1-320–607 expressed in bacteria to WT or 4M versions of the 263–281 peptide. (**b**) Binding of GST-ATG16L1-231–607 purified from yeast to WT or 4M versions of the 263–281 peptide. (**c**) Binding of GST-ATG16L1-231–607 purified from yeast or human cells (as indicated) to WT or 4M versions of the 263–281 peptide. The inset shows a magnified scanned image of an actual peptide microarray developed with the ligand purified from human cells. (**d**) Binding of an anti-biotin antibody to WT or 4M versions of the 263–281 peptide. (**e**) Binding of GST-ATG16L1-231–607 purified from human cells to the indicated mutant derivatives of the 263–281 peptide. (**f**) Binding of GST-ATG16L1-231–607 in T300 (T) or A300 (A) configurations (as indicated) purified from human cells to the wild-type 263–281 peptide (WT). Values are expressed as a fraction of the figures provided by the A300 ligand (*n*=54). (**g**) Background binding of GST-ATG16L1-231–607 (T300 or A300 variants, as shown) purified from human cells to the indicated irrelevant array positions. No subtraction of background signal was done in this case. Values are expressed as a fraction of the figures provided by the T300 ligand version. (**h**) Immunoblot analysis of pull-down assays (PD) between the indicated GST fusion proteins expressed in bacteria and full-length ATG16L1-T300 (T) or -A300 (A) (as indicated) present in crude bacterial lysates (left panel). The right panel shows an immunoblot assay for the presence of ATG16L1 (T or A) in the bacterial lysates before the assay was done (input, top), and a Coomassie-blue staining of a protein gel including the different GST fusion proteins (bottom). The 282–323 construct includes the non-functional part of the intracellular domain of TMEM59. All displayed results are representative of at least two repetitions.

We designed peptide microarrays in which the 19-amino acid peptide of TMEM59 that includes the ATG16L1-binding motif (263–281) was immobilized onto glass slides along with point mutants as controls. A subset of the peptides included a biotin group introduced at the end of the synthesis procedure to allow assessment of the amount of immobilized peptide. We probed the resulting arrays with a recombinant form of the WDD fused to GST and the HA tag (GST-HA-WD-320–607) purified from bacterial expression systems, or an extended WDD construct that includes residue 300 (GST-HA-ATG16L1-231–607, excluding the N-terminal and coiled-coil domains)^27^ produced in yeast. Both proteins were able to recognize the wild-type peptide but reacted poorly with the 4M derivative (Fig. 5a,b), suggesting that the observed interaction involves the WDD-binding motif. These results obtained with ligands produced in heterologous systems argue that the interaction with the target peptide is probably direct, since it is unlikely that a contaminating protein from bacteria or yeast (whose Atg16 ortholog lacks the WDD) mediates the binding event. Notably, a ligand preparation purified from mammalian expression systems showed superior binding activity (Fig. 5c), perhaps due to post-translational requirements provided by a native environment. Again, this interaction was motif-specific, since it was largely abrogated by mutation of the critical residues (4M version; Fig. 5c). None of the observed differences was due to dissimilar amounts of immobilized peptides, because an anti-biotin antibody reacted equally well with the relevant peptides (Fig. 5d). In addition, we detected reduced binding between GST-HA-ATG16L1-231–607 (produced in human cells) and peptides harbouring mutations in functionally important positions (Y268, Y277 and L280)^41^, but not in the case of mutants involving irrelevant residues such as V269, K273 or G278 (ref. 41; (Fig. 5e)). One notable exception was E272A, which showed normal binding to the WDD ligand (Fig. 5e) but, according to our previous studies, is critical for LC3 lipidation^41^. Interestingly, we also showed that this mutation does not inhibit recruitment of GFP-LC3 to TMEM59 (ref. 41), suggesting that E272 is exclusively involved in the LC3 lipidation step but not in recruitment of LC3-I to the lipidating complex^41^, a step known to be mediated by ATG16L1 through ATG5 and ATG3 (ref. 50). Therefore, an involvement of this residue in binding to ATG16L1 should not be expected in the peptide array setting. Together, these data argue that the observed interaction between the extended WDD ligand (residues 231–607) and the immobilized target peptides faithfully reflects the functional features of the 263–281 element.

We next used ligand preparations purified from human cells to test whether the T300A mutation alters the ability of the WDD to interact with the motif in this system. Binding assays conducted with GST-HA-ATG16L1-231–607 in a T300 or A300 configuration showed that the latter recognized the native peptide with a significantly lower affinity compared with the T300 form (Fig. 5f). The levels of background signal measured using a variety of methods were similar in the arrays developed with both ligand versions (Fig. 5g), indicating that the assay was performed under comparable conditions in both cases. Consistent with these results, pull-down studies carried out with fusion proteins between GST and the active element (GST-263–281; expressed and purified from bacteria) showed that full-length ATG16L1-A300 binds defectively to the target peptide (Fig. 5h). The interaction defect was more profound in this system compared with the data obtained using peptide microarrays (Fig. 5f), perhaps because the mutation alters ATG16L1 folding more severely in heterologous environments. Together, these data suggest that the T300A mutation causes an intrinsic structural alteration in the WDD that disrupts its ability to bind the ATG16L1-binding motif that we previously identified in TMEM59.

### Certain WDD-binding motif versions are insensitive to T300A

**Figure 6:**
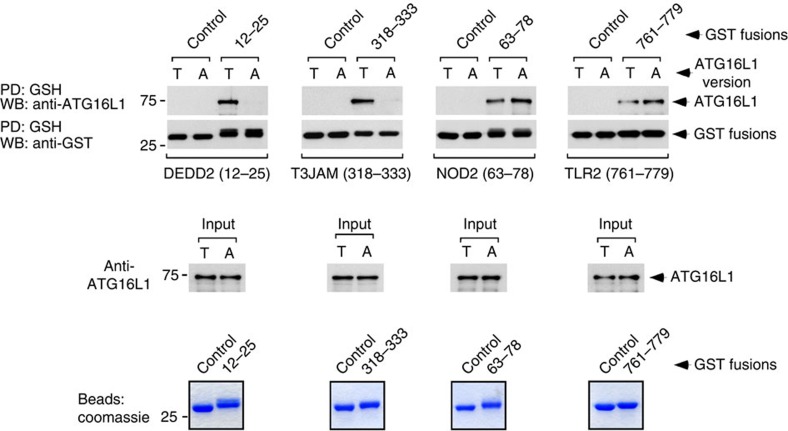
Differential impact of T300A on the interaction between ATG16L1 and different versions of the WDD-binding motif. Immunoblot analysis of pull-down assays (PD) between the indicated GST fusion proteins purified from bacteria and ATG16L1-T300 (T) or ATG16L1-A300 (A) present in crude bacterial lysates (top panels). The middle panels show immunoblot assays for the presence of ATG16L1 (T or A) in the bacterial extracts before the assay was carried out (input). The bottom panels show Coomassie blue-stained protein gels including the different purified GST fusion proteins used for the pull-down studies. The results shown are representative of at least three repetitions.

To determine whether this disability prevents binding of the WDD to alternative versions of the motif, we tested other peptides harbouring different configurations of the pattern for binding to both forms of ATG16L1. We previously identified four peptides of varying length present in unrelated proteins (DEDD2_12–25_, T3JAM_318–333_, NOD2_63–78_ and TLR2_761–779_) that are able to bind the WDD of ATG16L1 (ref. 41). Regardless of whether these elements play an ATG16L1-binding role in the context of their native proteins, when isolated they constitute alternative members of the ATG16L1-binding motif landscape^41^. Pull-down studies carried out with fusion constructs between GST and the different peptides showed that each was sufficient to bind ATG16L1-T300 expressed in bacteria (Fig. 6), thus confirming that they harbour an ATG16L1-binding signature. However, in two cases (DEDD2 and T3JAM), binding to the A300 form was blunted (Fig. 6), whereas the other two (TLR2 and NOD2) bound equally well (or better) to the CD risk protein (Fig. 6). Therefore, the disruption introduced by the T300A mutation in the ability of the WDD to bind its peptidic partners is restricted to particular versions of the ATG16L1-binding motif.

### Defective xenophagy by disruption of the TMEM59-ATG16L1 axis

**Figure 7:**
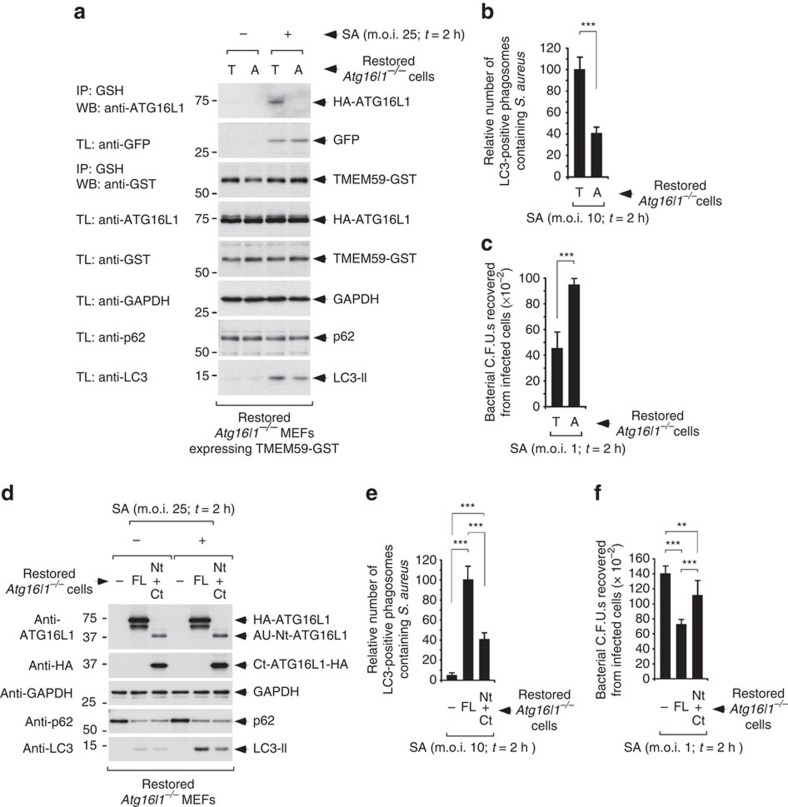
ATG16L1-A300 binds defectively to TMEM59-GST and impairs xenophagy in response to *S. aureus*. (**a**) Immunoblot analysis of GSH immunoprecipitates (IP) or total cell lysates (TL) of *Atg16l1*^−/−^ MEFs reconstituted with the indicated versions of ATG16L1 (T300 (T) or A300 (A)), retrovirally transduced with TMEM59-GST and subsequently infected (+) with a *S. aureus* strain (SA; 2 h; multiplicity of infection (m.o.i.)=25) that constitutively expresses GFP, or left uninfected (−). These results are representative of three repetitions. (**b**,**e**) Quantification of the number of LC3-positive phagosomes containing GFP-positive *S. aureus* bacteria 2 h after infection (m.o.i.=10) in *Atg16L1*^*−/−*^ MEFs restored with the indicated versions of ATG16L1 (T or A), full-length ATG16L1-T300 (FL), both ATG16L1 fragments that result from caspase-3 processing of the risk allele (Nt: 1–299; Ct: 300–607) or irrelevant vector (−). Values are expressed as a fraction of the scores obtained for cells expressing ATG16L1-T300 (mean±s.d. of triplicates; *n*=500 cells; ***P<0.001 Student's *t*-test). (**c**,**f**) Quantification of the colony-forming units (C.F.U.s) recovered from *Atg16L1*^*−/−*^ MEFs restored with the indicated versions of ATG16L1 (T or A) and infected with *S. aureus* for 2 h, m.o.i.=1 (mean±s.d., *n*=6, ***P<0.001, **P<0.01 Student's *t*-test). (**d**) Immunoblot analysis of endogenous LC3 activation and p62 expression levels induced by *S. aureus* infection (2 h, m.o.i.=25) of *Atg16l1*^−/−^ MEFs reconstituted with the indicated versions of ATG16L1 (full-length ATG16L1-T300 (FL) or both ATG16L1 fragments that result from caspase-3 processing of the risk allele (Nt: 1–299; Ct: 300–607)), or control vector (−). Results are representative of at least two repetitions.

We previously showed that, on infection with *S. aureus*, TMEM59 binds ATG16L1 to induce an unconventional autophagic event that promotes LC3 labelling of bacteria-containing phagosomes^41^. To test whether this interaction is altered by the T300A mutation, we carried out co-precipitation studies in cells infected with *S. aureus*. In this case we used *Atg16l1*^−/−^ MEFs restored with T300 or A300 versions of HA-ATG16L1, since they showed a stronger autophagic response to *S. aureus* compared with HCT116 cells (Supplementary Fig. 6). We found that, at early infection time points (2 h post-infection), TMEM59 associated defectively with ATG16L1-A300 compared with the T300 form (Fig. 7a). No major differences in infection rates were detected between the two cellular strains, as indicated by the levels of GFP (which is constitutively expressed by the bacteria) present in infected cells (Fig. 7a). Notably, we found no decay in the levels of full-length ATG16L1 (Supplementary Fig. 7) and no evidence of cleaved ATG16L1 or caspase-3 activation (Supplementary Fig. 7) in the same samples, arguing that the interaction defect does not require caspase-3-mediated processing of ATG16L1-A300.

The binding impairment displayed by the A300 allele correlated with reduced LC3 lipidation in response to *S. aureus* infection (Fig. 7a), decreased labelling of bacterial phagosomes with LC3 (Fig. 7b) and increased recovery of colony-forming units from infected cells (Fig. 7c), indicating that the T300A mutation causes defective xenophagy and reduced control of the infection at an early stage. As expected, *Atg16l1*^−/−^ MEFs showed poor xenophagy in reaction to the bacteria (Fig. 7d–f). Expression of non-risk ATG16L1 restored this response (Fig. 7d–f), part of which was lost in cells expressing separated N- and C-terminal ATG16L1 fragments (Fig. 7d–f). These data indicate a role of ATG16L1 in restraining *S. aureus* viability, and show that a portion of such activity is mediated by the WDD. Consistent with a function of TMEM59 in this pathway, depletion of this molecule using short interfering RNAs (siRNAs) recapitulated the xenophagic defects observed in the presence of ATG16L1-A300 or split ATG16L1 (Supplementary Fig. 8a–d). The cellular strains analysed did not show substantial differences in the number of intracellular GFP-positive bacteria (Supplementary Fig. 9), suggesting comparable infection rates. Together, these results point to the existence of an early xenophagic burst mediated by the WDD that is derailed by the A300 allele, at least in part through an inability of this mutant to bind the ATG16L1-binding motif. A fraction of such response is likely induced by TMEM59, although other molecules including the motif could also be involved. Importantly, cells expressing split ATG16L1 retained some xenophagic activity (Fig. 7d–f), suggesting that an overlapping layer of WDD-independent, conventional autophagy may also contribute to this function.

## Discussion

Since the discovery of the T300A polymorphism as a risk factor for CD (refs 6, 7), a substantial body of work has revealed a role of ATG16L1 in a variety of processes linked to intestinal homeostasis, including xenophagy against invading microorganisms^36^,^39^,^40^, trafficking of secretory vesicles in intestinal cells^36^,^38^ or the control of inflammation^34^,^35^. Additional work has demonstrated that many of these processes are also altered in the presence of the A300 allele^34^,^38^,^39^,^40^,^45^,^46^,^47^,^48^,^49^,^51^. The recent discovery that the T300A mutation increases susceptibility of ATG16L1 to caspase processing^46^,^47^ has provided a major advance towards identification of the molecular dysfunctions that cause these alterations. Thus, under stressful conditions such as bacterial infection or starvation, caspase-3 preferentially cleaves the risk allele and reduces the levels of the full-length protein, leading to impaired autophagy, decreased xenophagy and increased production of inflammatory mediators^46^,^47^.

We now reveal that the T300A mutation also derails the normal function of ATG16L1 in the absence of caspase-3-mediated cleavage. This single amino acid change alters the ability of the C-terminal WDD to interact with an amino acid motif that we previously identified in the transmembrane protein TMEM59 (ref. 41). In the context of this molecule, the mutation impairs binding between the WDD and the motif, a defect that results in poor autophagic labelling of the endosomes in which TMEM59 is located and slowed transit of these vesicles through the endocytic route. However, additional versions of the motif also displayed binding defects to the risk allele (see for example the motifs present in T3JAM and DEDD2; Fig. 6), suggesting that a cohort of ATG16L1 activators containing different configurations of the pattern have their activities blunted by the mutant protein in non-stressed conditions. Members of this family that may be involved in xenophagy (like TMEM59) could facilitate the onset of the uncontrolled inflammatory response that is typical of CD by being unable to restrain intracellular proliferation of the pathogens in the presence of the risk allele. However, our findings suggest alternative mechanisms through which ATG16L1-A300 could contribute to inflammation. For instance, expression of T3JAM and DEDD2 has been shown to increase c-Jun N-terminal kinase (JNK) signalling^52^ and apoptosis^53^, respectively, and both the JNK pathway^54^ and programmed cell death^55^ are thought to participate in intestinal inflammation. It is possible that an impaired interaction with ATG16L1-A300 (Fig. 6) could lead to decreased autophagic degradation and, as a consequence, increased levels of these mediators, thus promoting excessive activity of these signalling routes in the face of certain stimuli. Interestingly, we found that the interaction between the WDD and different motif versions is dissimilarly affected by the T300A mutation, arguing that some WDD-binding proteins might actually function better in the presence of the risk allele, or may not be affected at all. These results suggest a complex picture of the activity of the motif in the presence of ATG16L1-A300.

We show here that the A300 risk allele alters two cellular functions reminiscent of the defects observed in CD experimental model systems. First, ATG16L1-A300 impairs the normal intracellular trafficking of endosomes harbouring TMEM59. Derailed trafficking of secretory vesicles and a disorganized secretory compartment are well-known phenotypes of the intestinal Paneth and goblet cells of both mice and CD patients carrying the ATG16L1-A300 allele^38^,^46^. It is conceivable that a key regulator of these specialized vesicles also includes the motif and its dysfunction in the presence of the risk allele alters their proper secretory trafficking. Second, we found that ATG16L1-A300 derails an early WDD-dependent xenophagic response against *S. aureus* that contributes to fight the infection. Conflicting results have been published regarding the overall role of autophagy in *S. aureus* infection, ranging from no effect^56^ to a supporting role in bacterial replication^41^,^57^. Notably, we show here that ATG16L1 induces a concomitant layer of WDD-independent xenophagy in response to *S. aureus* (Fig. 7). A complex interplay between these two overlapping xenophagic activities, together with the varying intensity of basal, conventional autophagy in different cellular systems, and the known role of this process in favouring *S. aureus* replication at later stages^41^,^57^, might account for such different observations. In addition, ATG16L1 has been shown to promote tolerance to *S. aureus* α-toxin^56^, an activity that could be explained by the central function of autophagy in increasing resistance to stressful situations. But regardless the complexity of this particular system, the defects in WDD function caused by the T300A mutation that we describe here provide a novel mechanistic view to understand the more general alterations in xenophagy previously linked to this allele and CD (refs 39, 48, 49). Interestingly, the A300 polymorphism occurs at high frequency in the human population^6^,^7^, and it has been associated with additional physiopathological situations, like decreased survival following bone marrow transplantation^58^, improved survival in colorectal cancer^59^, gastric cancer susceptibility^60^ or Paget's disease of bone^61^. Therefore, understanding the biochemical consequences of the T300A mutation might be an important first step towards unravelling the physiological role of this allele beyond CD.

Our data support that the unconventional autophagic activity of WDD-binding proteins is also blunted in the presence of caspase-3-mediated cleavage of ATG16L1-A300, since TMEM59 was unable to trigger LC3 activation in a cellular system in which the entire pool of ATG16L1 was uncoupled in two fragments (thus mimicking caspase-mediated cleavage). In contrast, basal and rapamycin-induced autophagy were unaffected in these extreme conditions. This result is consistent with previous reports showing that the WDD is not involved in conventional autophagy^28^,^29^,^30^,^31^. However, other reports have found inhibition of the canonical pathway by cleaved ATG16L1 (refs 46, 47). These discrepancies suggest that, in some experimental systems, the WDD might play a role in conventional autophagy, or that under certain conditions the canonical route may include a relevant component of WDD-mediated unconventional autophagic processes similar to the one induced by TMEM59.

Our findings imply that a single amino acid change (T300A) is able to cause two different outcomes: altered affinity of the WDD for its target motif and increased susceptibility to caspase-3 processing. Although an alanine residue in position P1' of a consensus site is known to enhance caspase-3 processing of synthetic peptides^62^, the T300A mutation could also promote a shift in the overall structure of the WDD that might improve accessibility of the site to caspase 3. In this scenario, both effects could be the consequence of the same structural alteration imposed by the T300A mutation.

Together, our results help draw a coherent molecular model regarding how the A300 polymorphism causes pathology. We propose that a collection of motif-containing WDD-binding proteins have their natural autophagic activities modified by the risk allele under homeostatic situations in which levels of caspase 3 activity are negligible. In addition, the activity of these effectors would be blunted by ATG16L1-A300 processing in biological contexts in which stress promotes caspase-3 activation. Either way, alterations in the pathway involving motif-containing proteins and the WDD appear to be a prominent consequence of the ATG16L1-A300 polymorphism. Identification of additional members of this family of effectors will likely help unravel the signalling pathways whose dysfunction is required to trigger the onset of CD.

## Methods

### Cell lines and reagents

HEK-293T cells were obtained from the American Type Culture Collection. The HCT116 cell line derivatives KI-ATG16L1-T300A (generated by knock-in gene targeting) and *ATG16L1*^*−/−*^ have been described elsewhere^45^. MEFs deficient in *Atg16l1* have been previously described^36^. Cells were cultured at 37 °C and a humidified 5% CO_2_ atmosphere in DMEM (Invitrogen) containing 10% heat-inactivated FBS (Invitrogen) and 100 U ml^−1^ penicillin/streptomycin (Invitrogen). Bafilomycin, E64d, pepstatin, rapamycin, TNFα and cycloheximide were purchased from Sigma. zVAD.fmk was obtained from Calbiochem.

### DNA constructs

DNA constructs expressing HA-tagged human ATG16L1β (for mammalian and bacterial expression), HA-LC3, GFP-LC3, TMEM59 (full-length or Δ282, wild-type or 4M, untagged or C-terminal GST-tagged), p62-HA and CD16:7-263–281 have been previously described^41^. mRFP-LC3A was generated from the GFP-LC3A construct by replacing the GFP open reading frame with a PCR product encoding mRFP previously amplified from the mRFP-N1 plasmid (Addgene #54635). Cloning sites were Hind3/Pci1. TMEM59-GFP was built by inserting an in-frame PCR product encoding TMEM59 upstream GFP (Hind3/Pci1). ATG16L1 C- and N-terminal deletions were generated by PCR and insertion of the relevant amplicons downstream (Pci1/Not1) and upstream (EcoR1/Bsph1) HA and AU tags, respectively. GST fusions with the different peptides harbouring an ATG16L1-binding motif (TLR2_761–779_, DEDD2_12–25_, T3JAM_318–333_ and NOD2_63–78_) used for pull-down studies were constructed by PCR and subsequent insertion downstream, the GST open reading frame (EcoR1/Not1). ATG16L1-T300A, D299A, T300A/D299A and CD16:7-263–281-Y268A,E272A (2M) mutants were generated by site-directed mutagenesis (QuikChange, Stratagene). The sequences of the relevant oligonucleotides used to create these constructs are shown in Supplementary Tables 1 and 2. For expression in mammalian cells, constructs were cloned into the pEAK series of mammalian expression vectors or a retroviral derivative (P12-MMP)^63^ that includes internal ribosome entry site (IRES)-antibiotic resistance cassettes for selection in the appropriate selective media (see below). For expression in bacteria, GST fusion constructs were cloned into the pGEX vector (GE Healthcare) and ATG16L1 was expressed from the pET plasmid (Novagen). All constructs were verified by sequencing.

### Transfections and retroviral transductions

Overexpression and intracellular trafficking experiments were performed by transfection of the relevant DNA constructs using the jetPEI lipid reagent (Polyplus) following the manufacturer's instructions. KI-HCT116 showed lower transfection efficiency when compared to its parental counterpart; for this reason, many of the experiments were conducted in *ATG16L1*^−/−^ cells restored with T300 or A300 HA-ATG16L1. ATG16L1-deficient cells (MEFs and HCT116) were restored with the different ATG16L1 constructs by retroviral transduction. Retroviral transductions were also used to express TMEM59-GST in the indicated experiments. The relevant constructs were subcloned into P12-MMP vector versions including IRES-puromycin (to express full-length ATG16L1 or its N-terminal fragment) or IRES-hygromycin (to express the C-terminal fragment of ATG16L1 or TMEM59-GST) resistance cassettes that allow selection in puromycin (1 μg ml^−1^) and hygromycin (200 μg ml^−1^), respectively. Virus-containing supernatants were generated by co-transfecting 293T cells with the relevant P12-MMP constructs together with helper plasmids expressing gag-pol (pMD.gag-pol) and env (VSV-G; pMD-G). Infections were carried out by diluting the viral supernatants with fresh medium (1:1), and spinning the resulting mix onto the target cells for 1 h at 2,000 r.p.m., 32 °C.

### Cell lysis and co-immunoprecipitation assays

Cells were lysed in a buffer containing 1% Igepal CA-630 detergent (Sigma), 50 mM Tris HCl pH 7.5, 150 mM NaCl, 5 mM EDTA and protease inhibitors (Sigma). After a 5-min centrifugation step (4 °C), the resulting supernatants were evaluated for protein concentration (Bradford method, BioRad). For detection of endogenous LC3, t-ATG16L1 and t-casp 3, cells were lysed by resuspending the cell pellet in 2x standard sample buffer containing 4% SDS but lacking β-mercaptoethanol and bromophenol blue, followed by extensive boiling. β-mercaptoethanol and bromophenol blue were added to the samples after measurement of protein concentrations. For co-immunoprecipitations with GST-fusion constructs, total cell lysates were diluted to a final detergent concentration of 0.2% and incubated (4 °C, 1–3 h, rotation) with agarose beads coupled to glutathione (GE Healthcare). Beads were then washed at least three times with immunoprecipitation buffer and resuspended in 2x SDS sample buffer.

### Western blotting

Equal amounts of protein were resolved by SDS–polyacrylamide gel electrophoresis, transferred to a polyvinylidene-difluoride membrane (Millipore), and probed with specific antibodies against HA (monoclonal antibody (mAb), Babco MMS-101P, 1:1,000), AU1 (rabbit polyclonal, Babco PRB-130P, 1:1,000) or GFP (mAb, Babco MMS-118P, 1:1,000) tags, ATG16L1 (mAb, MBL M150-3, 1:1,000), tubulin (mAb, Sigma T4026, 1:40,000), GAPDH (mAb, Abcam ab8245, 1:10,000), LC3 (mAb, usually MBL M115-3, 1:1,000; MBL M186-3, 1:2,000, in Fig. 7 and Supplementary Fig. 8), human p62 (mAb, BD 610832, 1:1,000), mouse p62 (rabbit polyclonal, MBL PM045, 1:2,000), caspase 3 (rabbit polyclonal, Cell Signaling 9661, 1:1,000), GST (mAb, Santa Cruz sc-138, 1:1,000) or TMEM59 (rabbit polyclonal developed in-house)^41^. After incubation with appropriate secondary horseradish peroxidase-coupled antibodies (Jackson Immunoresearch), blots were developed by chemiluminescence (ECL system, Amersham). When re-probed, membranes were previously stripped for 15 min in a 7-M guanidinium hydrochloride (Sigma) solution. The LC3-II/LC3-I signal intensity ratio was calculated after using the Image J software to analyse the relevant scanned films. Uncropped scanned images of the most relevant western blots are shown in Supplementary Fig. 10.

### Pull-down assays

Isopropyl-β-D-thiogalactoside-induced bacteria (BL21) expressing the relevant GST fusion proteins (pGEX plasmid, GE Healthcare) were lysed by treatment with lysozyme (100 μg ml^−1^), freeze-thawing and sonication in a buffer containing 20 mM Tris HCl pH 8.0, 500 mM NaCl, 1 mM EDTA and 0.1% Triton X-100 (NET buffer). Lysates were cleared by centrifugation and incubated (1 h, 4 °C, rotation) with agarose beads coupled to glutathione (GE Healthcare). Loaded beads were washed extensively and used for ATG16L1 pull-down from induced crude bacterial lysates containing ATG16L1-T300 or -A300 proteins expressed from the pET plasmid (Novagen) in BL21(DE3) bacteria. Expression of these constructs in the soluble fraction was poor, but detectable. Beads were incubated with the amount of ATG16L1-containing lysate equivalent to 1 ml of induced culture (3 h, 4 °C, rotation), washed and processed for western blotting.

### Immunofluorescence studies and microscopy

Cells were seeded on poly-L-lysine coated coverslips, transfected, infected and/or treated the next day and fixed in 4% paraformaldehyde at the end of the experiment. Preparations were then permeabilized in a solution containing 0.5% Igepal CA-630 detergent, blocked in a solution containing 3% BSA and stained with the relevant primary antibodies for 1 h at room temperature. Endogenous LC3 was stained using a rabbit anti-LC3 polyclonal antibody (MBL PM036, 1:600) except for experiments involving infection with *S. aureus* where a mouse monoclonal anti-LC3 antibody (MBL M152-3 (IgG1 low affinity for protein A) 1:50) was used. Fluorocrohome-coupled secondary antibodies were purchased from Jackson Immunoresearch (Cy3) or Molecular Probes (Alexa-488). No immunofluorescence signal was detected in *Atg16l1*-deficient MEFs infected with *S. aureus*, indicating that the antibody is unable to directly recognize the bacteria (through protein A, naturally expressed by this strain) under the experimental conditions used. For experiments involving CD16:7-263–281 transmembrane chimera, cells were transfected with the indicated constructs, split the next day onto poly-L-lysine coated coverslips and stimulated 36 h post transfection with an anti-CD16 antibody (4 μg ml^−1^, mAb, NA/LE formulation, BD 555403) followed by 10 μg ml^−1^ rabbit anti-mouse Fcγ polyclonal serum (Jackson ImmunoResearch 315-005-008) to promote endocytosis. Aggregation was performed for 8 h. To stain for endocytosed CD16:7 chimeras, unpermeabilized cells were quenched *in vivo* with unlabelled goat anti-mouse κ chain antibody (Jackson ImmunoResearch 115-005-174, 60 μg ml^−1^, 40 min, 4 °C, 0.1% azide) to remove the cell surface signal provided by the aggregating antibody. Cells were then fixed, permeabilized and stained with a goat anti-mouse κ chain antibody conjugated to Cy3 (Jackson ImmunoResearch 115–165–174, 1:10,000). LC3 (both endogenous and transfected GFP-LC3) punctae quantification, TMEM59-GFP or CD16:7-263–281 vesicle counting and evaluation of GFP-expressing *S. aureus* in infected cells were conducted using a Zeiss Axiophot2 fluorescence microscope. Evaluation of endogenous LC3 punctae in cells transfected with TMEM59 was carried out by co-transfection with GFP and subsequent scoring of LC3 punctae (stained with a red fluorochrome; Cy3) only in GFP-positive cells. In cases where the relevant vesicles were accumulated in the perinuclear region (for example, TMEM59-GFP expressed in cells harbouring ATG16L1-T300) we inferred the number of vacuoles forming the irregular cluster by assessing the number of protruding lobuli. When indicated, samples were analysed under a Leica SP5 confocal microscope using the 488 (green), 561 (red) and/or 405 nm (blue) laser bands. Quantification of mRFP-LC3 intensity present on TMEM59-GFP vesicles was carried out over confocal images using the Intensity Quantification tool of the Leica Application Suite-Advance Fluorescence (LAS-AF) software.

### Peptide microarrays

Expression and purification of the recombinant ligand from bacteria (GST-HA-ATG16L1-320–607) was performed using conventional techniques. Briefly, the construct was cloned in the pGEX bacterial expression vector and induced in mid-log BL21 bacterial cells using 10 nM isopropyl-β-D-thiogalactoside at 25 °C for 8 h. Cells were lysed as described above (see ‘Pull-down assays') and the clarified supernatants incubated with GSH-agarose beads for 3 h at 4 °C. After extensive washing, the protein was eluted in 50 mM reduced GSH and concentrated by centrifugation using a YM-30 Centricon filtering device (Amicon). A typical yield was 100–150 μg of purified protein per litre of induced culture. The extended WDD form that includes residue 300 (ATG16L1-231–607) was unstable and poorly expressed in bacteria, as was full-length ATG16L1 (ref. 41). Expression and purification of the GST-HA-ATG16L1-231–607 construct from yeast cells was carried out using a modified version of the *Kluyveromyces lactis* Protein Expression Kit (New England Biolabs) where the protein was expressed without the secretion leader sequence included in the default vector (pKLAC2). Individual transformants were analysed for proper expression of the construct. The final cultures were grown to high density (OD600=10) and mechanically lysed with glass beads (425–600 μm, acid-washed, Sigma) and strong shaking provided by a FastPrep FP120 machine (BIO101 Savant) using 5 disruption cycles (5 s shaking, 1 min off between cycles, 4 °C) at a 4.0 power setting. Lysates were clarified by centrifugation (16,000*g*, 10 min, 4 °C) and the recombinant protein was recovered using GSH-agarose beads essentially as described above for expression in bacteria. To purify the recombinant ligands from mammalian cell lines, we used HEK-293T cells. Cells were transduced with retroviral constructs expressing the relevant ligands (GST-HA-ATG16L1-231–607, T300 or A300) followed by an IRES-puromycin element for selection (1 μg ml^−1^ of puromycin). Selected cultures were lysed in 1% Igepal CA-630 lysis buffer. The resulting clarified lysates were diluted to a final Igepal CA-630 concentration of 0.2% and incubated with GSH-agarose beads for 4 h at 4 °C. The recombinant protein was eluted from the washed matrix and concentrated as described above for the constructs expressed in bacteria. The approximate final yield was 1–2 μg per 10-cm plate (∼10^7^ cells). All samples were run in protein gels and stained with Coomassie blue to evaluate integrity and approximate protein quantity. A more accurate quantification was obtained using the DC Protein Assay kit (BioRad).

Peptide microarrays were ordered from the company JPT (Germany). All peptides were printed in triplicates in three different sub-arrays (nine spots per peptide in each microarray). Peptides were immobilized through the N-terminus using a long spacer (Tds), and some of them also included a biotin group between the spacer and the peptide to evaluate the amount of peptide printed in each spot. Slides were first blocked in a solution containing PBS/0.05% Tween-20 (PBS-T) supplemented with 5% BSA for 2 h at room temperature, and then washed in PBS-T with gentle shaking. Ligand incubation was performed in blocking solution containing the desired concentration of the recombinant protein (400 nM to 1 μM, as indicated in the relevant figure legends) for 2 h at room temperature, then overnight at 4 ° C. After washing, slides were incubated with a mixture of anti-HA (mAb; Babco MMS-101P) and anti-GST (rabbit polyclonal; Cell Signaling 2622) antibodies (10 μg ml^−1^ each, diluted in blocking buffer) for 2 h at room temperature. Arrays were then incubated with a solution containing a mixture of Cy5-labelled goat anti-mouse and anti-rabbit (Jackson ImmunoResearch) antibodies (1.5 μg ml^−1^ each in blocking buffer, 1 h at room temperature) followed by repeated washing in PBS-T. The anti-biotin antibody was purchased from Jackson ImmunoResearch (mAb, 200-002-211) and used at 10 μg ml^−1^ to directly probe the arrays, followed by incubation with a goat anti-mouse secondary antibody coupled to Cy5. A final wash with MilliQ distilled water allowed removal of salt residues. Slides were scanned using a GenePix 4000B slide scanner (Molecular Devices) and the resulting images analysed with the GenePix Pro software. The background signal provided by an irrelevant peptide present in the same array (FLAG) was subtracted from the relevant binding values before further processing the data for each peptide. Such background noise was typically 40–50 times lower than the specific signal.

### Bacterial strains and infection assays

Cells were infected with a GFP-expressing transformant of the *S. aureus* strain RN6390 (ref. 64) kindly provided by Dr A. Cheung. The indicated cell lines were incubated with the bacteria for 45 min, washed and treated with 100 μg ml^−1^ gentamicin for 1 h to inhibit extracellular proliferation of *S. aureus*. This was considered the 2 h time point at which cells were processed for further analysis. Determination of colony-forming units recovered from infected cells was done by lysing the infected cells in 0.1% Triton X-100 for 3 min and plating serial dilutions of the lysates in brain heart infusion (BHI)-agar plates for subsequent colony quantification.

### siRNA assays

MEFs were transfected with pre-designed pools of four RNA duplexes against mouse Tmem59 (On-TargetPlus, Dharmacon L-059473-01) using the transfection reagent DharmaFECT1 (Dharmacon) following the instructions provided by the manufacturers. Control siRNAs were a mix of duplexes having no perfect match with any mouse gene (On-TargetPlus Non-targeting pool, Dharmacon D-001810-10). Since Tmem59 expression levels are normally below the detection threshold of our antibody^41^, the degree of protein depletion caused by siRNA treatment was assessed after induction of Tmem59 expression by treatment with bafilomycin 48 h after transfection with the relevant siRNAs.

### Data availability

The data that support the findings of this study are available from the corresponding author on request.

## Additional information

**How to cite this article:** Boada-Romero, E. *et al*. The T300A Crohn's disease risk polymorphism impairs function of the WD40 domain of ATG16L1. *Nat. Commun.* 7:11821 doi: 10.1038/ncomms11821 (2016).

## Supplementary Material

Supplementary Information Supplementary Figures 1-10, Supplementary Tables 1-2
