# Supplementary material for: The T300A Crohn's disease risk polymorphism impairs function of the WD40 domain of ATG16L1
Source: Nat Commun. 2016 Jun 8;7:11821. doi: 10.1038/ncomms11821 (PMC4899871; doi:10.1038/ncomms11821)
Supplement: Supplementary Information — Supplementary Figures 1-10, Supplementary Tables 1-2 [file ncomms11821-s1.pdf]

SUPPLEMENTARY FIGURES

Supplementary Figure-1
(Pimentel-Muiños)

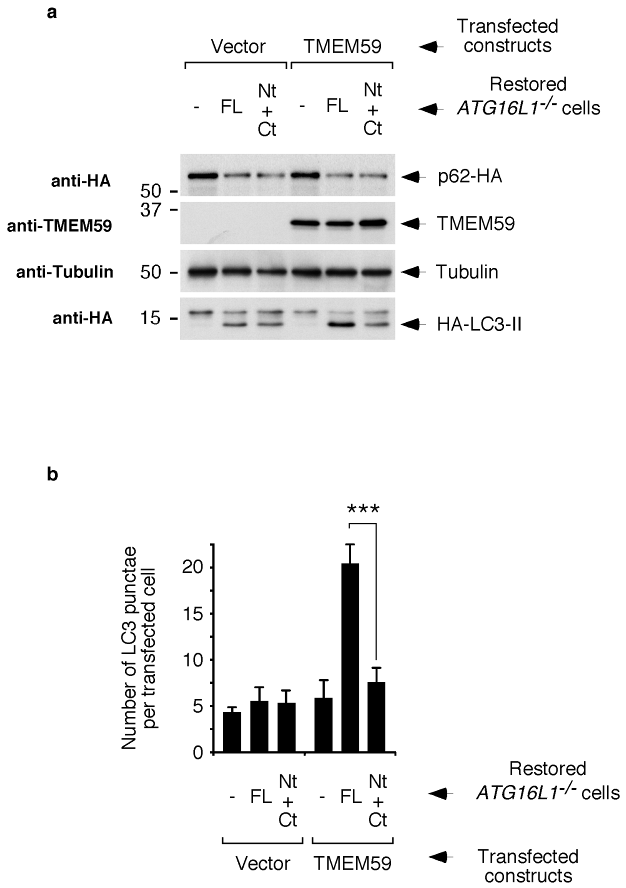

Supplementary Figure 1. Analysis of TMEM59-induced autophagy in restored *ATG16L1*$^{-/-}$ HCT116 cells. **(a)** Immunoblot analysis of co-transfected p62-HA degradation induced by TMEM59 overexpression for 36 h in *ATG16L1*$^{-/-}$ HCT116 cells restored with full-length ATG16L1-T300 (FL), the ATG16L1 fragments that result from caspase-3 processing of ATG16L1 (Nt: 1-299; Ct: 300-607) or irrelevant vector (-). **(b)** Quantification of endogenous LC3 punctae per transfected cell (evaluated only in cells positive for co-transfected GFP) induced by TMEM59 overexpression for 36 h in the same cell lines as in **a** (mean -/+ s.d.; n = 50 cells, ***$P < 0.001$ Student's *t*-test). The shown results are representative of two repetitions.

Supplementary Figure-2
(Pimentel-Muiños)

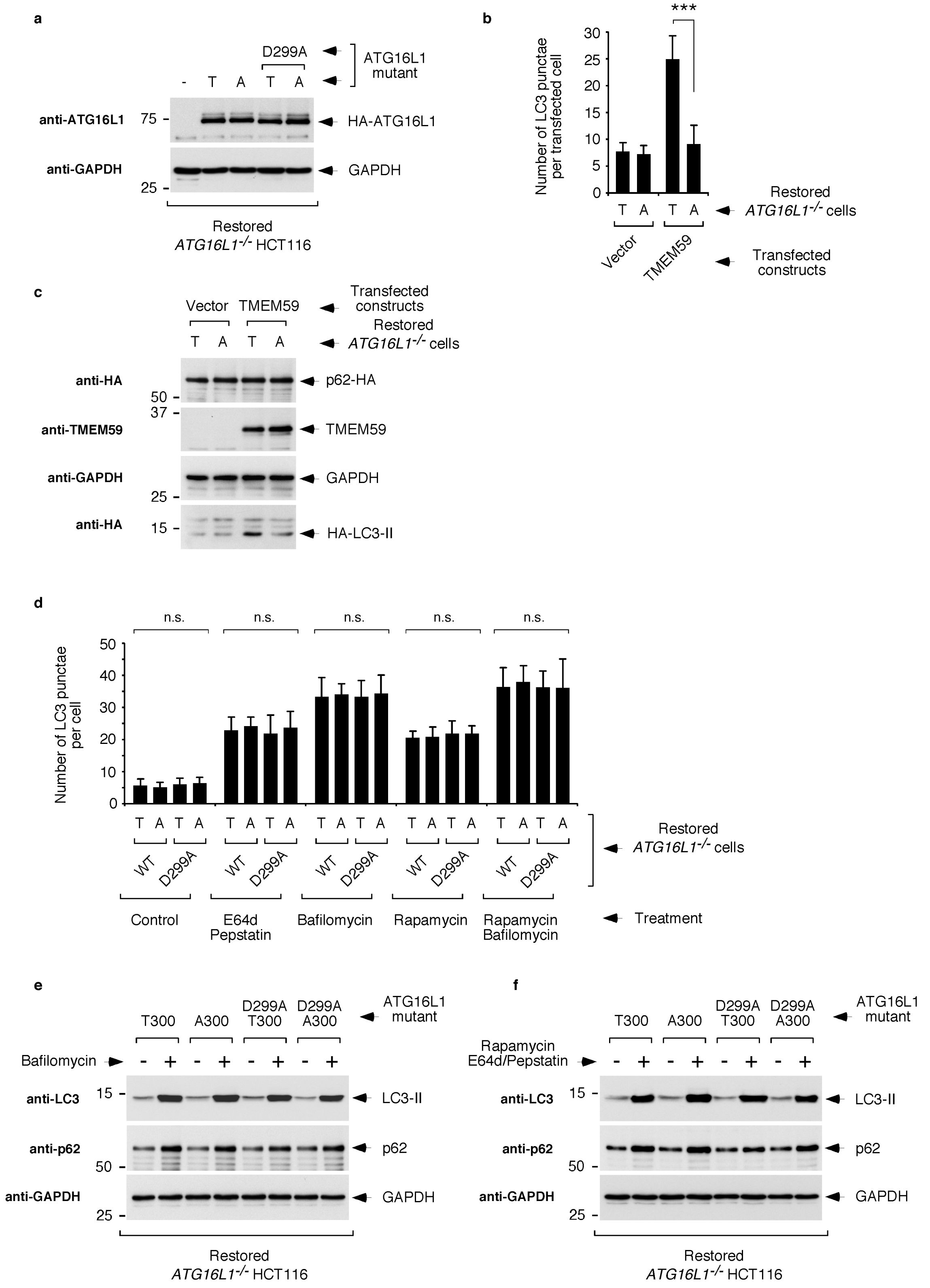

Supplementary Figure 2. Characterization of *ATG16L1*$^{-/-}$ HCT116 cells restored with mutated versions of HA-ATG16L1. **(a)** Immunoblot analysis of whole cell lysates obtained from *ATG16L1*$^{-/-}$ HCT116 cells restored with the indicated ATG16L1 constructs (T: HA-ATG16L1-T300; A: HA-ATG16L1-A300; T-D299A: HA-ATG16L1-T300-D299A; A-D299A: HA-ATG16L1-A300-D299A), or with control vector (-). **(b)** Quantification of endogenous LC3 punctae per transfected cell (evaluated only in cells positive for co-transfected GFP) induced by TMEM59 overexpression for 24 h in the indicated cell lines previously analyzed in **a**. Shown are mean values -/+ s.d. (n = 50 cells, ***P < 0.001 Student's *t*-test). **(c)** Immunoblot analysis of co-transfected p62-HA expression levels induced by TMEM59 transfection for 24 h in the same restored *ATG16L1*$^{-/-}$ HCT116 cells shown in **b**. **(d)** Quantification of endogenous LC3 punctae per cell induced in the cellular strains described in **a** in response to the indicated stimuli (50 nM bafilomycin for 8 h; 10 μg/ml E64d/pepstatin for 8 h and 2 μg/ml for rapamycin for 8 h; mean -/+ s.d.; n = 50 cells). None of the minor differences observed for the same treatment between cell lines were significant (n.s., not significant, P > 0.05 Student's *t*-test). All differences observed for the same cell line between control and treated samples were statistically significant (not shown, P < 0.001 Student's *t*-test). **(e,f)** Immunoblot analysis of endogenous LC3 lipidation and p62 expression levels in the same cell lines as in **d** subjected to the shown treatments. Treatment conditions were as in **d**. All results are representative of at least two repetitions.

Supplementary Figure-3
(Pimentel-Muiños)

a

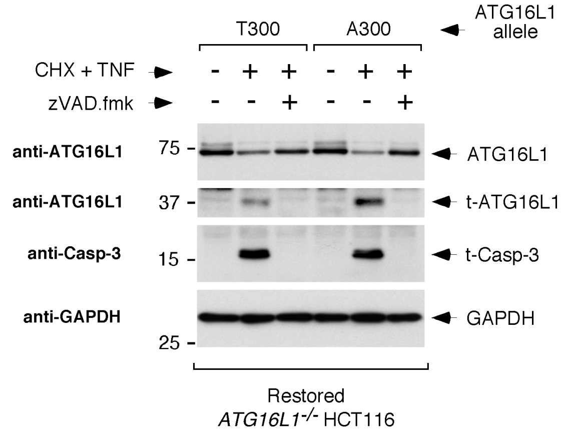

b

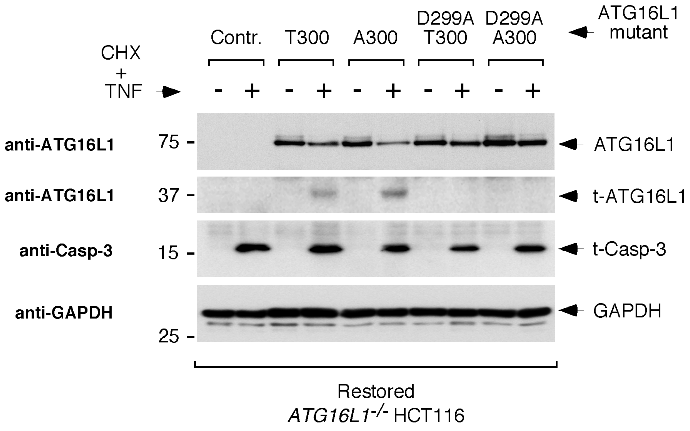

Supplementary Figure 3. Inhibition of caspase-mediated cleavage of ATG16L1 by zVAD.fmk or mutation of the caspase 3 target site in ATG16L1. (a,b) Immunoblot assays of whole cell lysates from *ATG16L1*$^{-/-}$ HCT116 cells restored with the indicated ATG16L1 constructs subjected to the shown treatments (TNF, 20 ng/ml; CHX, 10 μg/ml; **a,** zVAD.fmk, 50 μM) for 8 h. The displayed images are representative of two repetitions.

a

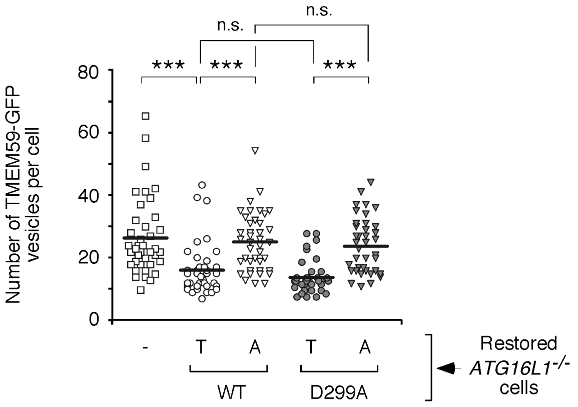
n.s.
n.s.
***
***
***
80
60
40
20
0
Number of TMEM59-GFP vesicles per cell
-
T
A
T
A
WT
D299A
Restored
ATG16L1-/-
cells

b

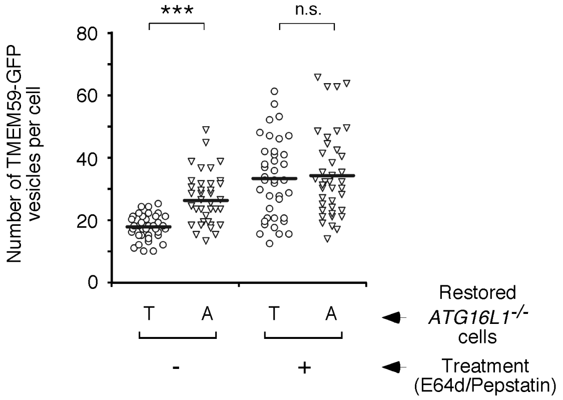
***
n.s.
80
60
40
20
0
Number of TMEM59-GFP vesicles per cell
T
A
T
A
Restored
ATG16L1-/-
cells
-
+
Treatment
(E64d/Pepstatin)

Supplementary Figure 4. TMEM59-GFP trafficking defects do not require ATG16L1-300A caspase cleavage and are normalized by lysosomal inhibitors. **(a,b)** Scatter plots showing quantifications of the number of TMEM59-GFP positive vesicles per cell after transfection of this construct into *ATG16L1*$^{-/-}$ HCT116 cells reconstituted with the indicated ATG16L1 constructs or irrelevant vector (-); (n = 40 cells, ***$P < 0.001$ Student's *t*-test, n.s., not significant, $P > 0.05$ Student's *t*-test). Cells in **a** were fixed 24 h post-transfection. Cells in **b** were treated with E64d/pepstatin (10 μg/ml each) for the last 8 h of culture and fixed 32 h after transfection. Results are representative of two repetitions.

Supplementary Figure-5
(Pimentel-Muiños)

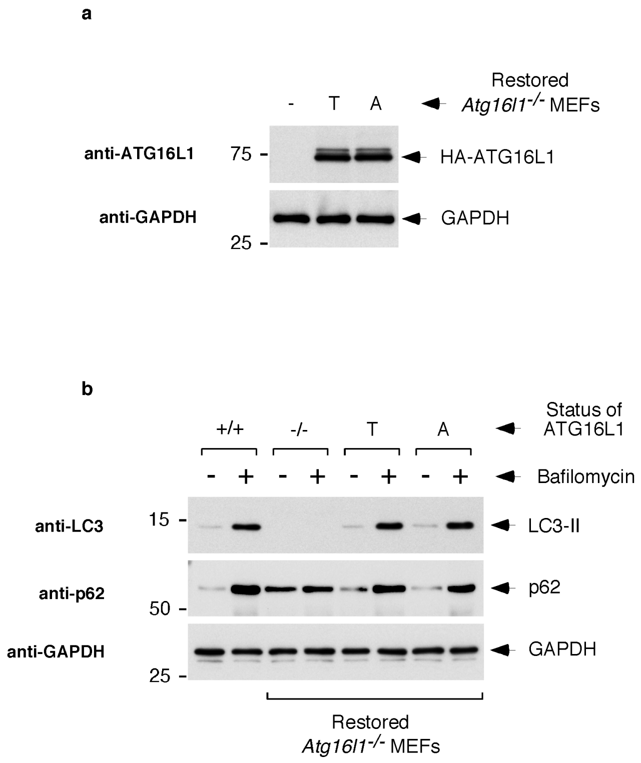

Supplementary Figure 5. Characterization of *Atg16l1$^{-/-}$* MEFs reconstituted with T300 or A300 versions of HA-ATG16L1. (a) Immunoblot analysis of total cell lysates from *Atg16l1$^{-/-}$* MEFs cells restored with the indicated ATG16L1 constructs (T: HA-ATG16L1-T300; A: HA-ATG16L1-A300), or empty vector (-). **(b)** Immunoblot analysis of the autophagic response (lipidation of endogenous LC3 and p62 expression levels) of wild-type MEFs (+/+) and the same cellular strains shown in **a** subjected to bafilomycin treatment (50 nM, 8 h). The displayed Western-blot images are representative of two repetitions.

Supplementary Figure-6
(Pimentel-Muiños)

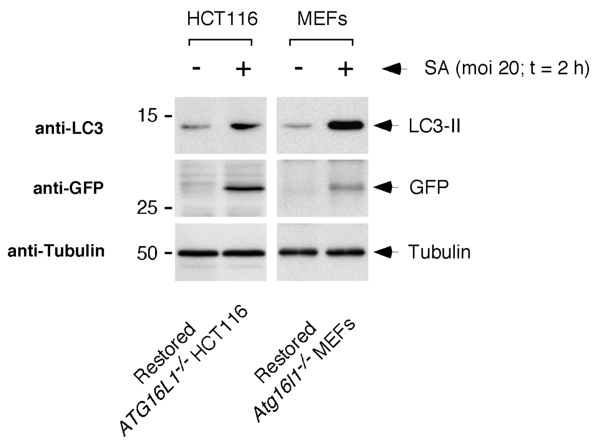

Supplementary Figure 6. MEFs display stronger LC3 activation in response to *S. aureus* compared with HCT116 cells. Immunoblot analysis of total cell lysates from the indicated ATG16L1-deficient cell lines retrovirally restored with HA-ATG16L1-T300 and subjected to infection with *S. aureus* (2 h; moi = 20).

Supplementary Figure-7
(Pimentel-Muiños)

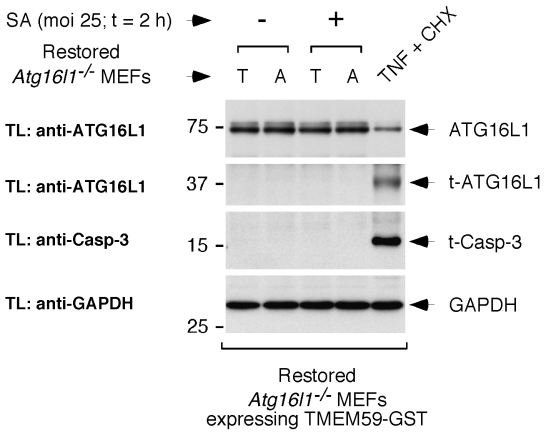

Supplementary Figure 7. Absence of ATG16L1-A300 and caspase-3 cleavage in cells infected with *S. aureus* at early times post-infection. Immunoblots of total cell lysates (TL) from the same samples shown in Fig. 7a run in parallel with a positive control for cleaved ATG16L1 (t-ATG16L1) and caspase 3 (t-Casp-3). This control sample was derived from *Atg16l1*$^{-/-}$ MEFs restored with HA-ATG16L1-A300 and subjected to TNF (20 ng/ml) and CHX (10 μg/ml) treatment for 8 h. Images displaying t-ATG16L1 and t-Casp-3 immunoblots show long exposure times. These results are representative of two repetitions.

Supplementary Figure-8
(Pimentel-Muiños)

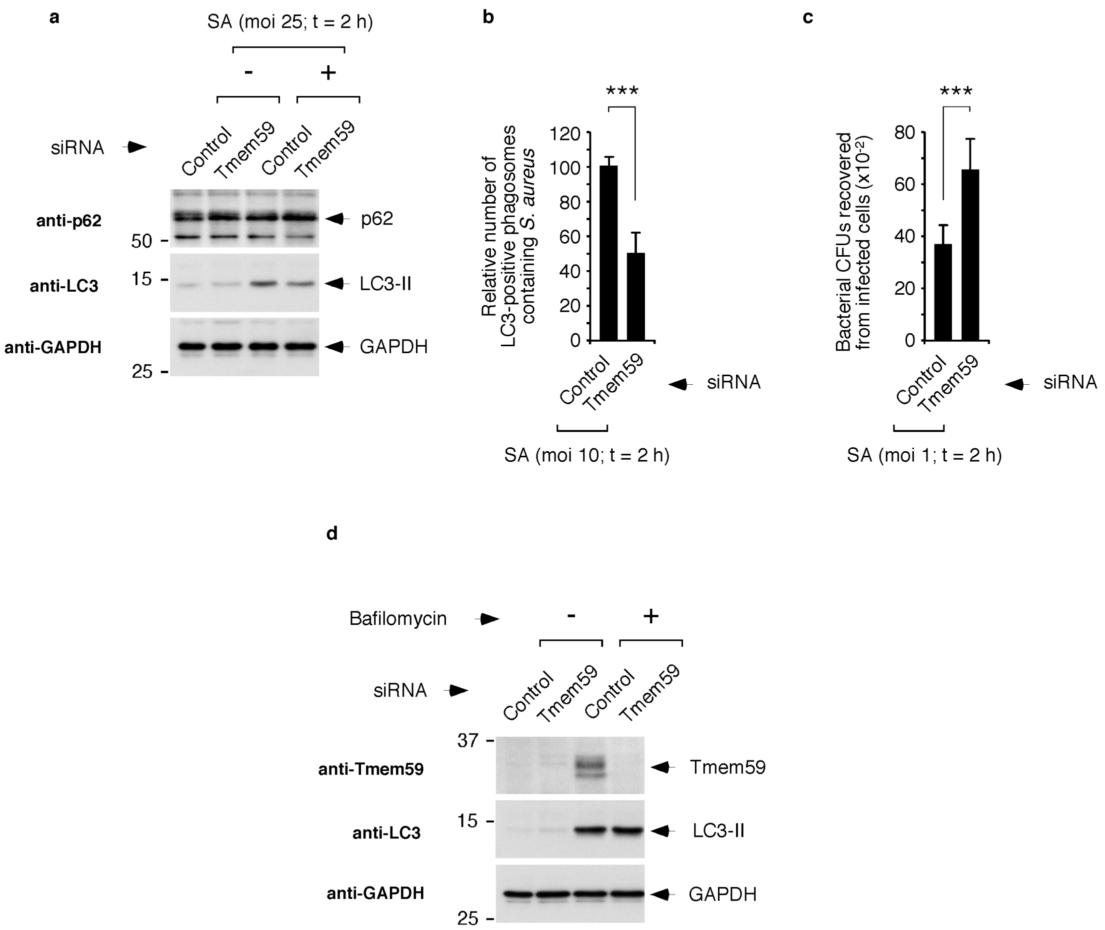

Supplementary Figure 8. Role of TMEM59 in xenophagy against *S. aureus* at early infection times. **(a)** Immunoblot analysis of endogenous LC3 activation and p62 expression levels in response to *S. aureus* infection (2 h, moi = 25) of *Atg16L1*$^{-/-}$ MEFs restored with ATG16L1-T300 and transfected with control siRNAs or siRNAs against Tmem59 (as indicated). Bacterial infection was done 48 h after siRNA transfection. **(b)** Quantification of the number of endogenous LC3-positive phagosomes containing *S. aureus* bacteria (GFP-positive) 2 h after infection (moi = 10) of *Atg16L1*$^{-/-}$ MEFs restored with ATG16L1-T300 and transfected with the same siRNA species and under the same conditions as in **a**. Values are expressed as a fraction of the scores obtained for cells transfected with control siRNAs (mean -/+ s.d. of triplicates; n = 500 cells; ***$P < 0.001$ Student's *t*-test). **(c)** Quantification of the colony forming units (CFUs) recovered from *Atg16L1*$^{-/-}$ MEFs restored with ATG16L1-T300, transfected with the same siRNAs and under the same conditions as in **a**, and subsequently infected with *S. aureus* for 2 h (moi = 1) (mean -/+ s.d., n = 6, ***$P < 0.001$ Student's *t*-test). **(d)** Immunoblot analysis of Tmem59 depletion in MEFs transfected with control siRNAs or siRNAs against Tmem59 (as indicated) and treated 48 h later with bafilomycin (8 h, 50 nM). Treatment with bafilomycin was carried out to prevent lysosomal degradation of Tmem59 and induce the expression levels of the protein above the detection limit of the antibody, thus allowing evaluation of siRNA-mediated knockdown efficacy. All results shown in this figure are representative of two repetitions.

Supplementary Figure-9
(Pimentel-Muiños)

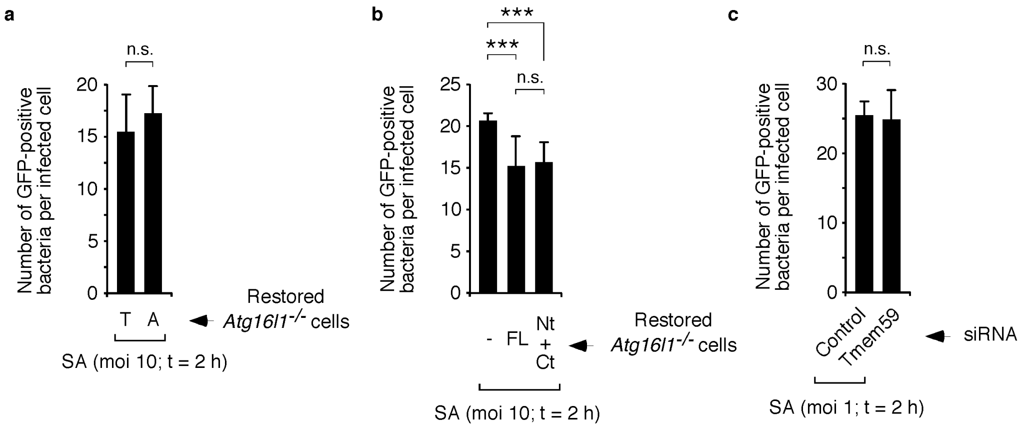

Supplementary Figure 9. Similar *S. aureus* infection rates in MEFs restored with different ATG16L1 versions or depleted for Tmem59 expression. The immunofluorescence slides evaluated in Figs. 7b, 7e and Supplementary Fig. 8b were scored for the number of GFP-expressing *S. aureus* bacteria per infected cell. Shown are mean values -/+ s.d. (n = 120 cells; n.s., not significant, $P > 0.05$ Student's *t*-test; ***$P < 0.001$ Student's *t*-test).

Supplementary Figure-10
(Pimentel-Muiños)

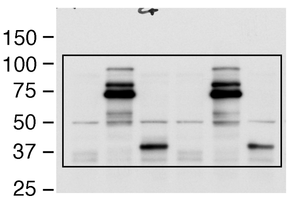

Fig. 1a (anti-ATG16L1)

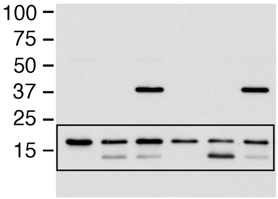

Fig. 1a (anti-HA)

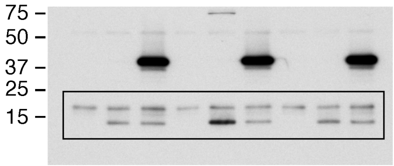

Fig. 1b (anti-HA)

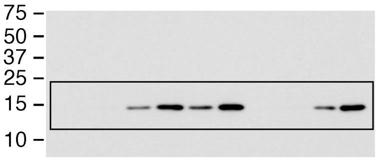

Fig. 1d (anti-LC3)

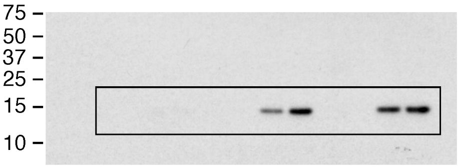

Fig. 1e (anti-LC3)

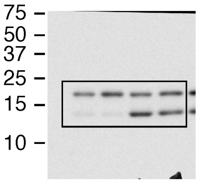

Fig. 2b (anti-HA)

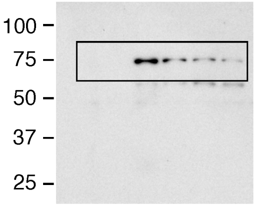

Fig. 4a (anti-AU)

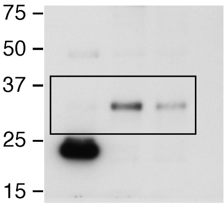

Fig. 4b (anti-TMEM59)

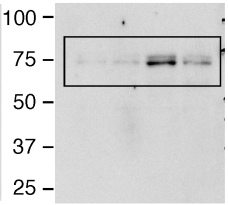

Fig. 4c (right-
anti-ATG16L1)

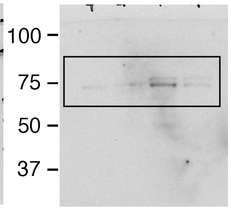

Fig. 4c (middle-
anti-ATG16L1)

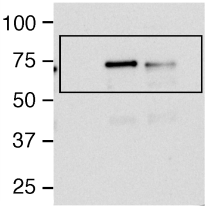

Fig. 4c (left-
anti-ATG16L1)

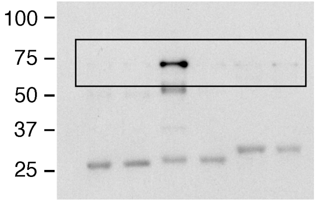

Fig. 5h (anti-ATG16L1)

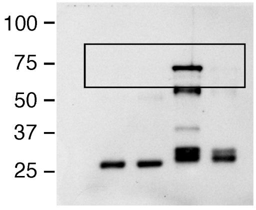

Fig. 6 (DEDD2-
anti-ATG16L1)

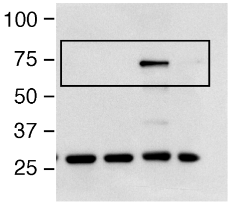

Fig. 6 (T3JAM-
anti-ATG16L1)

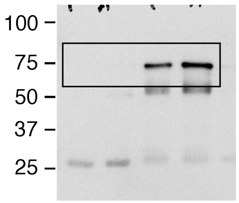

Fig. 6 (NOD2-
anti-ATG16L1)

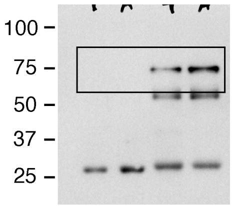

Fig. 6 (TLR2-
anti-ATG16L1)

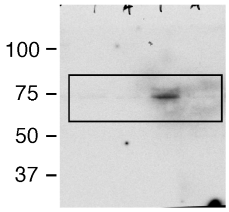

Fig. 7a (anti-
ATG16L1)

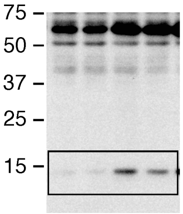

Fig. 7a (anti-
LC3)

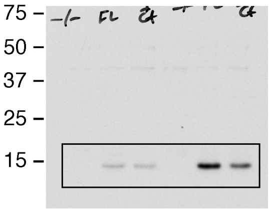

Fig. 7d (anti-LC3)

Supplementary Figure 10. Uncropped scanned images of the most relevant immunoblots displayed in the indicated figures.

SUPPLEMENTARY TABLES

Supplementary Table 1. Oligonucleotides for PCR

ATG16L1-Nt (1-299)-Pci-fw	5' gggcccacatgtcgtcgggcctccgcgccgctg 3'
ATG16L1-Nt (1-299)-Not1-Stop-rev	5' cccggggcggccgctttaatccacattgtcctgggggactgggaag 3'
ATG16L1-Ct (300-607)-EcoR1-Kozak-fw	5' gggcccgaattcgccaccatgggcactcatcctggttctggtaaagaagtgag 3'
ATG16L1-Ct (300-607)-BspH1-rev	5' cccgggtcatgaggtactgtgcccacagcacagctttgc 3'
mRFP-Hind3-Kozak-fw	5' gggcccaagcttgccaccatggcctcctccgaggacgtcatcaag 3'
mRFP-Pci1-rev	5' cccgggacatgtagctgcccccggcgccggtggagtggcggccctcggcg 3'
TMEM59-Hind3-Kozak-fw	5' gggcccaagcttgccaccatggcggcgccgaaggggagcctc 3'
TMEM59-Pci1-rev	5' cccggggcacatgttaatttcagaatgagcaagattcac 3'
TLR2 (761-779)-EcoR1-fw	5' gggcccgaattctacctggagtggcccatggacgaggctc 3'
TLR2 (761-779)-Not1-Stop-rev	5' cccggggcggccgctttatctcagatttacccaaaatccttcccg 3'
DEDD2 (12-25)-EcoR1-fw	5' gggcccgaattctgggaggaggatgagtgcctggactac 3'
DEDD2 (12-25)-Not1-Stop-rev	5' cccggggcggccgctttacgacagcatcccgtagtagtccaggcac 3'
T3JAM (318-333)-EcoR1-fw	5' gggcccgaattctggaggagccagtatgaggctctgaag 3'
T3JAM (318-333)-Not1-Stop-rev	5' cccggggcggccgctttacccaagggtcctccagtcctccttcag 3'
NOD2 (63-78)-EcoR1-fw	5' gggcccgaattctgggaggtcctctcctgggaggactac 3'
NOD2 (63-78)-Not1-Stop-rev	5' cccggggcggccgctttagcccaggaggtggaagccctcgtagtc 3'

Sequences of the oligonucleotides used to generate new DNA constructs by PCR.

Supplementary Table 2. Oligonucleotides for site-directed mutagenesis

ATG16L1-T300A-TOP	5' caggacaatgtggatgctcatcctggttctgg 3'
ATG16L1-T300A-BOTTOM	5' ccagaaccaggatgagcatccacattgtcctg 3'
ATG16L1-D299A-TOP	5' cccccaggacaatgtggccactcatcctggttctg 3'
ATG16L1-D299A-BOTTOM	5' cagaaccaggatgagtggccacattgtcctggggg 3'
ATG16L1-T300A-D299A-TOP	5' cccccaggacaatgtggccgctcatcctggttctgg 3'
ATG16L1-T300A-D299A-BOTTOM	5' ccagaaccaggatgagcggccacattgtcctggggg 3'
CD16:7-263-281-E272A-TOP	5' cagtatgttccctctgccaagctgagtatctatg 3'
CD16:7-263-281-E272A-BOTTOM	5' catagatactcagcttggcagagggaacatactg 3'
CD16:7-263-281-E272A,Y268A (2M)-TOP	5' acagctgtggagcaggctgttccctctgccaag 3'
CD16:7-263-281-E272A,Y268A (2M)-BOTTOM	5' cttggcagagggaacagcctgctccacagctgt 3'

Sequences of the oligonucleotides used to generate new DNA constructs by site-directed mutagenesis.
